# Cost-effectiveness analysis of universal varicella vaccination in Turkey using a dynamic transmission model

**DOI:** 10.1371/journal.pone.0220921

**Published:** 2019-08-13

**Authors:** Lara J. Wolfson, Vincent J. Daniels, Matthew Pillsbury, Zafer Kurugöl, Cuneyt Yardimci, Jeffrey Kyle, Ener Cagri Dinleyici

**Affiliations:** 1 Center for Observational and Real-World Evidence (CORE), Merck & Co., Inc., Kenilworth, New Jersey, United States of America; 2 Department of Pediatrics, Faculty of Medicine, Ege University, Izmir, Turkey; 3 Medical Affairs, MSD Turkey, Istanbul, Turkey; 4 Department of Pediatrics, Faculty of Medicine, Eskisehir Osmangazi University, Eskisehir, Turkey; Erasmus MC, NETHERLANDS

## Abstract

**Background:**

In 2013, Turkey introduced one-dose universal varicella vaccination (UVV) at 12 months of age. Inclusion of a second dose is being considered.

**Methods:**

We developed a dynamic transmission model to evaluate three vaccination strategies: single dose at 12 months (1D) or second dose at either 18 months (2D-short) or 6 years of age (2D-long). Costs and utilization were age-stratified and separated into inpatient and outpatient costs for varicella and herpes zoster (HZ). We ran the model including and excluding HZ-related costs and impact of exogenous boosting.

**Results:**

Five years post-introduction of UVV (1D), the projected varicella incidence rate decreases from 1,674 cases pre-vaccine to 80 cases/100,000 person-years. By 25 years, varicella incidence equilibrates at 39, 12, and 16 cases/100,000 person-years for 1D, 2D-short, and 2D-long strategies, respectively, using a highly effective vaccine. With or without including exogenous boosting impact and/or HZ-related costs and health benefits, the 1D strategy is least costly, but 2-dose strategies are cost-effective considering a willingness-to-pay threshold equivalent to the gross domestic product. The model predicted a modest increase in HZ burden during the first 20–30 years, after which time HZ incidence equilibrates at a lower rate than pre-vaccine.

**Conclusions:**

Our findings support adding a second varicella vaccine dose in Turkey, as doing so is highly cost-effective across a wide range of assumptions regarding the burden associated with varicella and HZ disease.

## Introduction

The varicella-zoster virus (VZV) is a herpes virus that causes the pruritic rash of chickenpox (varicella), most commonly in childhood, and the painful blistering rash known as herpes zoster (HZ) or shingles, mainly a disease of older or immunocompromised individuals. The virus remains latent for life in dorsal root ganglia of infected individuals when not activated [1].

In countries such as the United States (US) that have adopted universal varicella vaccination (UVV) in childhood, the incidence of varicella has fallen substantially [2]. Nonetheless, there are concerns that UVV, by reducing levels of circulating wild-type VZV, may reduce exogenous boosting, the proposed phenomenon whereby continuous virus exposure may enhance protective cell-mediated immunity, thereby reducing the risk of VZV reactivation [3]. A decrease in exogenous boosting could potentially cause an increased incidence of HZ and/or shift the incidence of natural varicella to older age groups, who experience more severe illness, greater complications, and greater hospitalization rates, hence greater costs, than younger individuals.

The clinical importance and magnitude of exogenous boosting are not well-characterized, however, and evidence for the effect of UVV on exogenous boosting and the incidence of HZ is inconclusive [4,5]. Reports from the US, where 2-dose UVV has been in place since 2006, suggest that the risk of HZ is reduced among children vaccinated for varicella [6,7]. Instead, some disease models predict an increase in HZ after instituting effective varicella vaccination programs if exogenous boosting is assumed to occur [8,9]. Prior modelled estimates for high-income countries (pre-2014) that included UVV impact on HZ via reduction or absence of exogenous boosting found that routine childhood varicella vaccination can lead to incremental cost-effectiveness ratios (ICERs) exceeding commonly accepted thresholds [9]. These findings highlight the importance of considering numerous factors when developing varicella vaccination models.

Factors important in developing varicella vaccination models include potential impacts of herd immunity, age-specific varicella incidence rates, the interactions between varicella and HZ, and the most important consideration for modelling effects of varicella vaccination, namely, vaccine efficacy when used in the real-world setting, i.e., vaccine effectiveness. A recent meta-analysis concluded that all varicella vaccines are equally effective; however, that study primarily focused on OKA-strain vaccines and found insufficient information to analyze time since vaccination, an important determinant of vaccine effectiveness [2]. Instead, results of a study of varicella outbreaks in Germany indicate there may be differences in effectiveness among vaccine brands [10], and another recent meta-analysis found differences by vaccine brand in the rate of breakthrough varicella [11]. Moreover, in a recently published large-scale study in Taiwan, the relative risk of breakthrough varicella was shown to differ by vaccine manufacturer among OKA-strain vaccines [12].

In Turkey, varicella vaccines have been available in the private sector since 2000, and one-dose UVV at 12 months of age was implemented in 2013, with current coverage reported to be >95%for children 12–36 months of age [13]. An ongoing prospective multicenter study, VARICOMP, has provided important epidemiologic and economic surveillance data on varicella-related hospitalizations among children in Turkey for the 5 years between 2008 and 2013 (pre-vaccine era) and 2013 and 2018, during which time breakthrough varicella requiring hospitalization was observed most commonly in previously healthy children at 5 years after a single vaccine dose [14,15]. Other reports of breakthrough varicella in Turkey have been published as well. In one report from 2008–2009, among vaccinated children, 28% experienced breakthrough varicella, mostly mild disease but moderate to severe for one-quarter of these children; the risk of breakthrough varicella was determined to be 3.7-fold higher among children vaccinated ≥5 years vs. < 5years earlier [16]. More recently, in April 2016, a varicella outbreak occurred at three preschools despite 62% of children having received one-dose vaccination [13].

Inclusion of a second dose of varicella vaccine at 18 months or 6 years is now being considered in Turkey to minimize outbreaks, especially in children <1 year, and to decrease breakthrough varicella, hospitalization rates, and burden of disease [2,17]. The aim of this study was to evaluate the costs and benefits of different UVV schedules and vaccine options employing a dynamic transmission model for Turkey. We investigated results by varying assumptions of vaccine effectiveness. In addition, we evaluated the impact of including or excluding HZ-related costs and exogenous boosting on conclusions regarding cost-effectiveness.

## Methods

### Model overview

We developed for this study a mathematical model of varicella virus transmission and the occurrence of varicella and HZ in an age-structured population, using a model structure similar to that employed previously by other investigators [18–21]. The model supports one- and two-dose varicella vaccination schedules, as well as catch-up programs (e.g., second dose at first opportunity or campaign strategies). Here we describe the model and its application in a cost-effectiveness analysis of these strategies using UVV for the age-structured population in Turkey.

A full description of the model can be found in S1 Appendix.

### The demographic model

The demographic model describes how persons enter, age, and exit the simulated population of the larger varicella transmission model. It is similar to the initial-boundary-value problem for age-dependent population growth described in more detail by Hethcote in 1997 [22].

The demographic model divides the population into 47 discrete age groups to allow health and economic outcomes to be stratified by age, and to address public policy questions involving realistic varicella vaccination programs (Table A in S1 Appendix). The age groups are 0–1 month, 1–6 months, and 6–11 months, followed by age groups defined to support typical vaccination schedules for the first varicella dose (i.e., 1-month age groups from 11–24 months), the second varicella dose (1-year age groups from 2–20 years), and detailed estimations of health and economic outcomes for adults (5-year age groups from 20–70 years and 10-year age groups thereafter). The model employs country-specific information for population size, fertility, and all-cause mortality rates by age group, which is accessible from the United Nations (UN) Population division [23].

### Structure of the epidemiologic model

**Fig 1:**
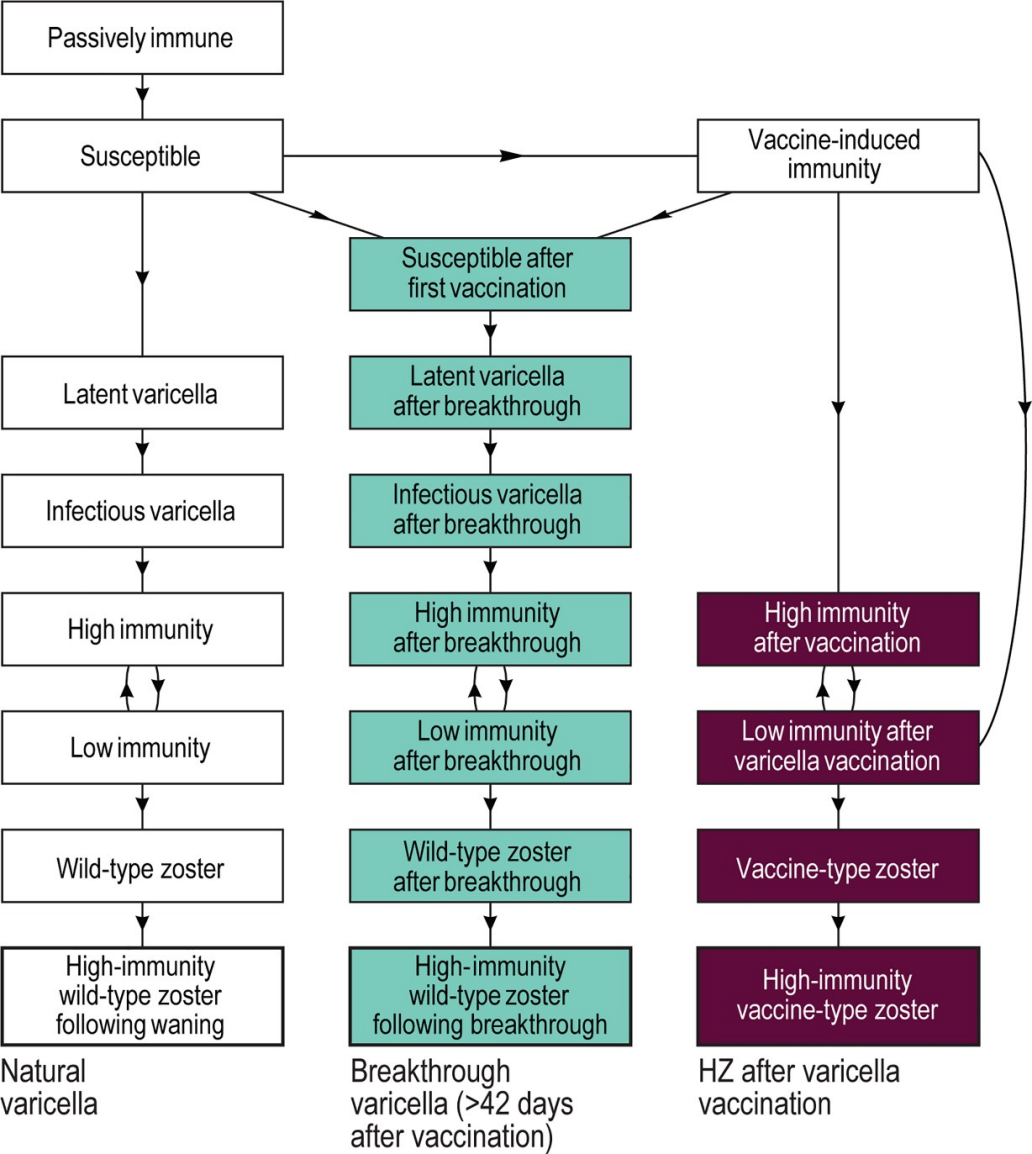
Overview of varicella dynamic transmission model structure. The left column of compartments represents the natural history and evolution of natural varicella through wild-type HZ (zoster). The other two columns represent the natural history following varicella vaccination with the center column flowing from breakthrough varicella through wild-type HZ after breakthrough and the right column flowing from varicella vaccinated directly to vaccine-type HZ (without breakthrough varicella). For simplicity and clarity we have omitted HZ vaccination compartments and transitions as well as transitions to death. A more detailed set of diagrams can be found in S1 Appendix including detailed compartment transitions and rates as well as both varicella and HZ vaccination (Figs A–D in S1 Appendix). HZ, herpes zoster.

The epidemiologic model is a deterministic compartmental model based on extensions of the work of Schuette and Hethcote [18] and van Hoek et al [21] (Fig 1). The epidemiologic outcomes of the model are determined by interactions between varicella and HZ, allowing the model to be used to investigate outcomes and costs of both varicella and HZ as well as varicella alone. The natural history of disease, and associated disease outcomes, can occur due to infection with natural varicella and wild-type HZ, or varicella vaccination followed by breakthrough varicella infection and wild-type HZ, or varicella vaccination followed by vaccine-type HZ, as illustrated in Figs A–D in S1 Appendix.

Persons enter the population at birth either without natural immunity, or with natural maternal immunity that wanes over time, depending on the fraction of births to mothers with antibodies to pass on to their children. There is also the possibility of receiving one or two doses of varicella vaccine with a time- and age-dependent probability that is determined by the parameters of the varicella vaccination program being simulated.

After the first dose of varicella vaccine, possible outcomes include full protection (high immunity after vaccination), partial protection (low immunity after vaccination), and vaccine failure (susceptible after first vaccination), in which case those individuals are treated as if they never received the vaccine at all. Protection after varicella vaccination can wane from full to partial protection with subsequent development of breakthrough varicella. The second dose of varicella vaccine follows a similar pattern except it is assumed that there are no second-dose vaccine failures.

Persons who become infected with either natural or breakthrough varicella spend 14 days, after which they recover in either 7 or 4.5 days, for natural or breakthrough varicella respectively [18]. Following varicella infection, the virus remains latent and can later reactivate as HZ. The model assumes that infectious individuals become permanently immune to varicella after clearing the infection, including those who may have been previously vaccinated with any number of varicella vaccine doses.

Individuals can develop HZ at age-dependent reactivation rates, which also vary with their disease history in order of descending likelihood as follows: (1) they had natural varicella and were never effectively vaccinated (wild-type HZ following natural varicella), (2) they were varicella vaccinated and developed breakthrough varicella (vaccine-type HZ following breakthrough varicella), and (3) they were varicella vaccinated and never developed breakthrough varicella (vaccine-type HZ). Protection after HZ vaccination can wane from full to partial protection with subsequent development of breakthrough HZ (breakthrough HZ following HZ vaccination). The model assumes that HZ occurs only once.

The model allows for modulating the rate at which individuals who are not vaccinated and susceptible to HZ activation become protected from HZ through capture of an exogenous boosting effect. The exogenous boosting effect is assumed to be proportional to the varicella force of infection (FOI) and to last for 20 years [8]. The proportionality constant may be age dependent; however, in the current study we assume the effect is equal to FOI for all ages. Although there is some evidence that this hypothesis may not be a realistic representation [4,5,20,24,25], it is the base case if exogenous boosting is turned on in the model.

The FOI for each age group is the sum of the product of disease transmission rate between age groups and infectious population density of each age group. The transmission rate with people in each age group with those in other age groups (mixing matrix) is country-specific and is determined during model calibration. In the current study we assume proportionate mixing, i.e., mixing is proportional to activity level in the age groups and each age group is equally likely to mix with all others.

### Vaccination strategies and parameters

For each vaccination strategy in the model, the vaccine characteristics are probability of vaccine failure, take (the degree to which a vaccine confers full protection from disease), and waning (the rate at which vaccinated people become susceptible to varicella infection again) for each dose [26]. The vaccine parameters chosen for the base model were derived by van Hoek et al. [21] using published data from a 10-year follow up study of Varivax (Merck & Co., Inc., Kenilworth, NJ, USA) for both one and two doses [27] and are defined in Table C in S1 Appendix together with disease-specific parameters used in the model. Thus, the varicella vaccine-related properties shown in the table are specific to assuming the use of the Varivax or ProQuad (Merck & Co., Inc., Kenilworth, NJ, USA) vaccines, as the two vaccines, both containing MSD OKA-strain varicella, are considered to be immunologically equivalent [28]. Scenarios including HZ vaccination are not analyzed in the current paper, but the parameterization of the HZ vaccine is included in Table C in S1 Appendix.

For the base model, we evaluated three vaccination strategies as compared with no vaccine: (1) a single dose at 12 months of age (1-dose), (2) a second dose administered at 18 months of age (2-dose-short), and (3) a second dose administered at 6 years of age (2-dose-long). Both of the 2-dose strategies incorporate a 1-dose strategy for the first 5 years before the introduction of the second dose, i.e., the existing 1-dose strategy in Turkey since 2013.

Coverage for the first varicella vaccine dose was defined as the percentage of eligible individuals who received the first dose (base case 95%, range 75% to 95%), with administration at an age of 12 months. Coverage for the second dose was defined as the percentage of those individuals, who after receiving the first dose also received the second dose (base case 90%, range 70% to 95%), with second-dose administration at 18 months (at the same visit as other vaccine booster doses) or at 6 years (at the same visit as MMR and TDaP-IPV).

In addition to the different vaccination strategies, we ran the model both including and excluding exogenous boosting. As noted above, the model includes exogenous boosting assuming full temporary immunity [8,20,21]. We calibrated the model assuming the probability of being boosted from exposure to circulating varicella is 100% of the varicella FOI and adjusted the age-dependent HZ reactivation rates to fit the observed pre-vaccine HZ incidence.

### Economic model

The model supports detailed input of resource utilization and both direct and indirect costs. Varicella-related utilization and direct costs are separated into inpatient and outpatient categories, which thereby includes both the complicated and uncomplicated cases by factoring in the proportion of patients assumed to seek care for varicella, and the cost per patient of seeking that care, as a weighted average across both complicated and uncomplicated cases. The HZ-related utilization and direct costs include both uncomplicated HZ and postherpetic neuralgia (PHN; Table D in S1 Appendix). Most costs and utilization parameters are age stratified.

The economic model combines the quality-adjusted life-years (QALYs) decrement from perfect health with an estimate of life-years lost attributable to varicella to produce an overall metric for health outcomes, for which model inputs include health utility values by age for natural varicella, breakthrough varicella, HZ, vaccine-type HZ, and PHN. The values used in the base model are defined in Table E in S1 Appendix, as sourced from the work of Littlewood et al. [29].

### Model calibration

The model is calibrated to fit observed pre-vaccine-era varicella seroprevalence. We use an empirical contact matrix [30], and an age specific susceptibility that is fitted to the seroprevalence. These contact rates and susceptibilities are then used to estimate the transmission matrix. Assuming that the HZ contribution to FOI is very small compared with varicella disease contribution, we then independently calibrated to HZ incidence by adjusting HZ reactivation rates. Details of the calibration process are provided in S1 Appendix.

### Model outcomes and software

The model supports a variety of outcomes expressed using 1 year as the unit of time. The key outcome of the model is the complete numerical solution to the differential equations describing the each compartment’s population as a function of time (provided in S1 Appendix). In addition we provide a set of aggregate outcomes that are described below.

The health outcomes generated by the model include the following incidence categories each comprising several subcategories:

- Varicella incidence, comprising natural, breakthrough, and congenital varicella
- HZ incidence, comprising wild-type and vaccine-type HZ
- HZ incidence, comprising uncomplicated HZ and PHN
- Deaths, comprising varicella-related and HZ-related deaths
- Varicella vaccine doses, comprising (if included) first, second, and catch-up doses
- HZ vaccine doses, comprising first doses (if included)

For each incidence category above, the sum of all subcategories gives the total incidence. The HZ incidence category has two different stratifications.

The model was implemented using the *Mathematica* software package, 11.3 (Wolfram Research, Champaign, IL, USA).

### Sensitivity analyses

We performed both one-way and probabilistic sensitivity analyses. The sensitivity analyses reported here are restricted to variations in economic parameters and in parameters related to vaccine properties.

We performed a one-way sensitivity analysis, varying vaccine properties (degree of protection against infection and against disease, given a breakthrough infection), vaccination strategy, costs, utilization, and QALY input parameters to examine the total costs avoided (savings) and the QALY loss avoided by the 2-dose-short program (vaccination at 12 months and 18 months) as compared with no vaccination or one-dose vaccination. For most variables, we assumed a ±20% variation. For percentages, we chose upper limits based on the smaller of 100% and +20% and for lower limits the larger of 0% and -20%. For health utilities, we enforced the limits of their range (0 to 1). For values that were already on the boundary, we used the base case value as one of the boundaries and applied some estimate of the other boundary.

Probabilistic sensitivity analysis (PSA) was performed by randomly selecting a large set of cost, utilization, coverage, and vaccine input parameters from the appropriate distributions and determining the effect on these variations on outcomes. We considered 1000 sets of parameter value variations drawing from the distributions defined in Table F in S1 Appendix, including only the 1-dose and 2-dose-short programs with exogenous boosting and including HZ costs and outcomes. We used the gamma distribution for representing uncertainty in cost parameters because costs are non-negative and highly skewed [31] and uncertainty in median duration of protection, a parameter ranging from 0 to infinity.

We used a beta distribution to represent uncertainty in quality of life (QoL) parameters because utilities or health-related QoL weights in this analysis are assumed to be between 0 and 1. In addition, we used beta distribution for representing uncertainty in vaccine uptake parameters because vaccine uptake in the model was represented by a proportion of persons being vaccinated.

The properties of vaccines include degree of protection against infection, and degree of protection against disease given a breakthrough infection. The degree of protection is 1 minus residual susceptibility (i.e., relative risk of infection among vaccine and placebo recipients). Because the relative risk ranges from zero to infinity, a log-normal distribution is used [31].

Further details are reported in S1 Appendix.

### Data sources for Turkey

Turkey-specific data used in the model can be found in S2 Appendix tables and figures as noted below.

Population-specific initialization parameters were used to calibrate the model, including age distribution of the population, fertility, pre-vaccine varicella seroprevalence by age, and zoster incidence by age. Data for fertility and population distribution were sourced from the 2017 United Nations (UN) World Population Prospects database files containing number of births by age of mother for Turkey [32] and the population distribution by age [33].

Turkish seroprevalence data were drawn from two pre-vaccine era studies: one that screened children 1 to 16 years of age in eastern Turkey in late 2003 [34] and one that screened participants older than 15 years old in Izmir, Turkey, between 2009 and 2010 [35]. Both studies used an enzyme-linked immunosorbent assay (ELISA) for VZV-specific IgG. Since the model assumes a static population, it will produce a monotonically increasing seroprevalence profile; therefore, we approximate the seroprevalence data using a monotonically increasing function (S2 Appendix, Table A). S2 Appendix, Table B depicts the age-specific susceptibilities resulting from calibration of Turkey seroprevalence.

The Turkish case fatality rates were derived from a combination of data from the VARICOMP study covering children up to 15 years old [14] and from a study in Brazil for the older ages [36]. We made the assumption that the case fatality rate for breakthrough varicella is one-fifth of the natural varicella case fatality rate (S2 Appendix, Table C).

Furthermore, for the Turkey calibration we have assumed there is no endogenous boosting and that all contacts with varicella infectives that would be sufficient to cause varicella can boost to full temporary immunity from HZ reactivation (S2 Appendix, Table D), an assumption similar to that made in other models [8,21]. Data for Turkey regarding the epidemiology of HZ were lacking; therefore, we drew on an Israeli study [37] and calculated the simple mean incidence rate for ages ≥65 years to determine rates where reported age group structure differed from ours as used in the model (S2 Appendix, Table E).

The economic inputs were drawn from a variety of sources. Whenever possible, Turkey-specific costs were used. All costs are expressed in 2015 Turkish lira (TRY), and costs that were not originally in TRY were converted using the 2015 currency conversion, with 1 USD = 2.834 TRY [38]. Cumulative costs were calculated in 2015 TRY using a 100-year time horizon, discounted at 3% annual rate, from the societal perspective. Costs and utilization, most drawn from Turkish data sources, were age stratified and separated into inpatient and outpatient costs for varicella and uncomplicated and PHN-related costs for HZ (S2 Appendix, Table F). All the strategies considered are in S2 Appendix, Table G. The cost for each vaccine dose was 60.55 TRY (±10%) including vaccine administration. At the time of our study, the Turkish vaccination schedule included visits at 12 months, 18 months, and 6 years, so no additional vaccination-related visits were foreseen in any of the modeled vaccination scenarios [39].

The one-way sensitivity input parameters for Turkey are listed in S2 Appendix, Table H.

### Alternative vaccines considered

For the purposes of this analysis, we considered three types of varicella-containing vaccines, in line with current monovalent varicella vaccine formulations available in the Turkish market. The "highly effective" vaccine was defined based on the properties of Varivax (MSD OKA-strain), as described in van Hoek et al. [21] and drawing on clinical trial results from Kuter et al. [27]. The "moderately effective" vaccine was based on the properties of Varilrix (GSK OKA-Strain; GlaxoSmithKline, Brentford, London, UK), as described in three recent modelling papers [40–42] without reanalysis of the clinical trial data [43]; the duration data are sourced from a separate real-world evidence study (original reference not publicly available, cited in Ouwens et al. [40]). Thus, for the highly and moderately effective vaccines, we used published parameterizations widely reported in the literature. Finally, the "weakly effective" vaccine is based on properties of Suduvax (Green Cross MAV/O6-strain; GC Pharma, Gyeonggi-do, Republic of Korea), as described in four published papers [44–47]. Model parameters for each of these three varicella vaccine types are summarized in Table 1.

**Table 1:** Properties of common varicella vaccine parameters used in the analyses.

		Dose	Highly effective vaccine	Moderately effective vaccine	Weakly effective vaccine
Failure	P		4%	5%	25%^b^
Take	T_1_	1^st^	100% (93–100%)	65.4% (57.2%–72.1%)	55%^c^
	T_2_	2^nd^	100% (97–100%)	94·9% (92·4%–96·6%)	70%^d^
Waning	1/𝜔_𝜐1_	1^st^	25 years (15–67)^a^	17 years (NR)	5 years^e^ (NR)
	1/𝜔_𝜐2_	2^nd^	77 years (38–200)^a^	77 years (NR)	15 years^f^ (NR)
Cost			60.55 TRY	57.96 TRY	55.36 TRY

NR, ranges not reported; TRY, Turkish lira.

^a^Limits according to van Hoek et al. [21].

^b^Based on seroconversion rates of 76.7% [44] and 74% [43].

^c^Conservative assumption based on calculations from Oh et al. [44], yielding unadjusted direct effectiveness of 59% (-82%, 91%); data on MAV-strain vaccines from a case-control study estimating effectiveness of -5% (-61.9%-31.9%) [42], and an estimate of 13.0% of all varicella vaccines in South Korea [43].

^d^Assuming a roughly similar relationship between one- and two-dose effectiveness as for the moderately effective vaccine.

^e^An exponential curve fit to data from Choi et al. [43] would have estimated a waning rate of 1.44183 years; 5 years was taken as a conservative assumption.

^f^Assuming a 1:3 relationship between waning rates of 1^st^ and 2^nd^ dose as for the highly effective vaccine

## Results and discussion

### Model calibration

**Fig 2:**
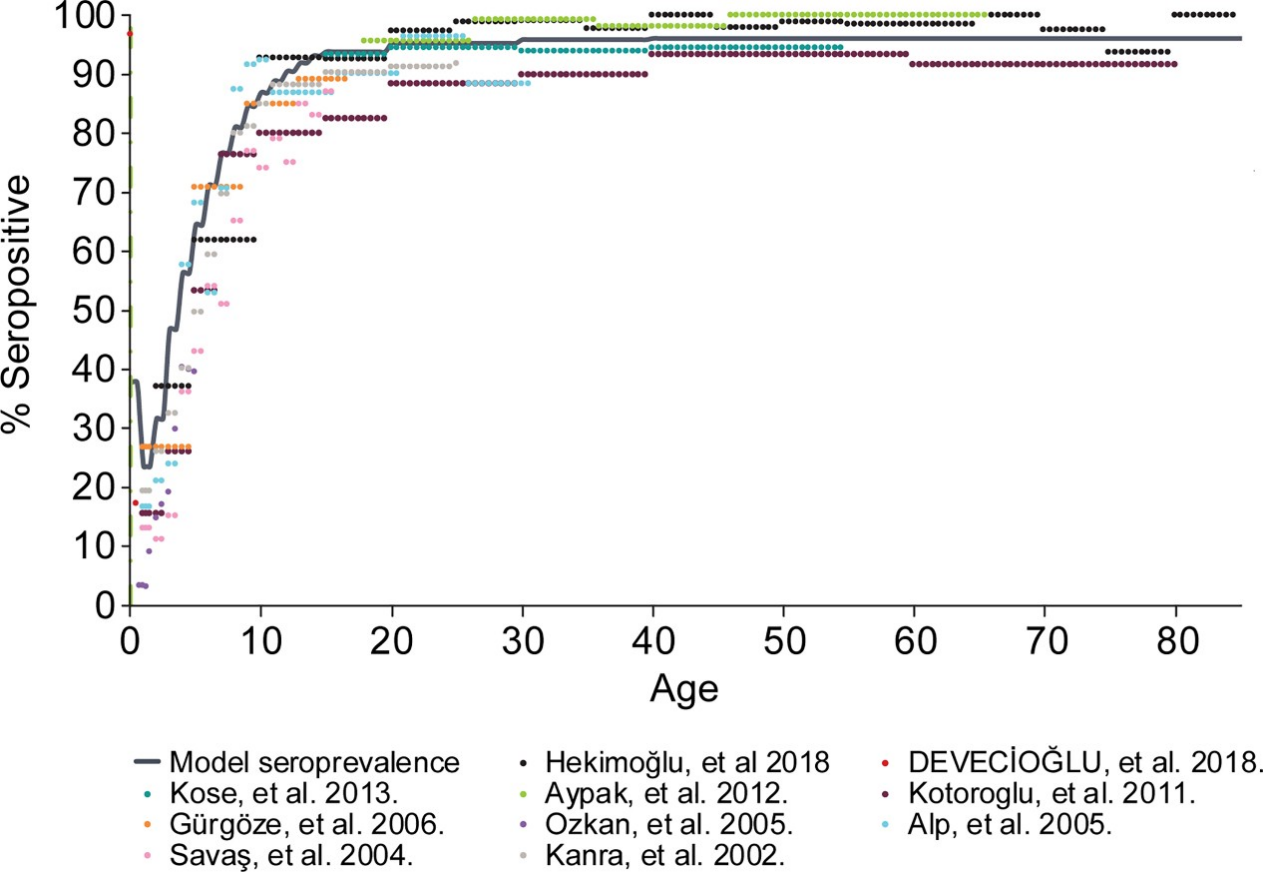
Model calibration: Varicella seroprevalence fit. The figure depicts the results of the model calibration to pre-vaccine varicella seroprevalence data, smoothed by fitting with a shifted sigmoid function and then by adjusting age-specific probabilities of acquiring an infection during a single contact with an infectious person, whereby a good fit was obtained with regard to change in incidence by age and with prior studies as well [34,35,48–55].

The model was calibrated using population-specific initialization parameters including age distribution of the population, fertility, varicella seroprevalence by age, and zoster incidence by age, as described above. Fig 2 depicts the results of the model calibration to pre-vaccine varicella seroprevalence data, smoothed by fitting with a shifted sigmoid function (provided in S2 Appendix, Table A), and then by adjusting age-specific probabilities of acquiring an infection during a single contact with an infectious person, whereby a good fit was obtained with regard to change in incidence by age and with prior studies as well [34,35,48–55]. The modeled HZ incidence rate and the adjusted observed incidence rate (provided in S2 Appendix, Table E) are depicted in S1 Fig.

### Varicella incidence by vaccine strategy and vaccine effectiveness

In this section we restrict ourselves to the scenarios that include exogenous boosting since the varicella incidence is not significantly affected by exogenous boosting.

**Fig 3:**
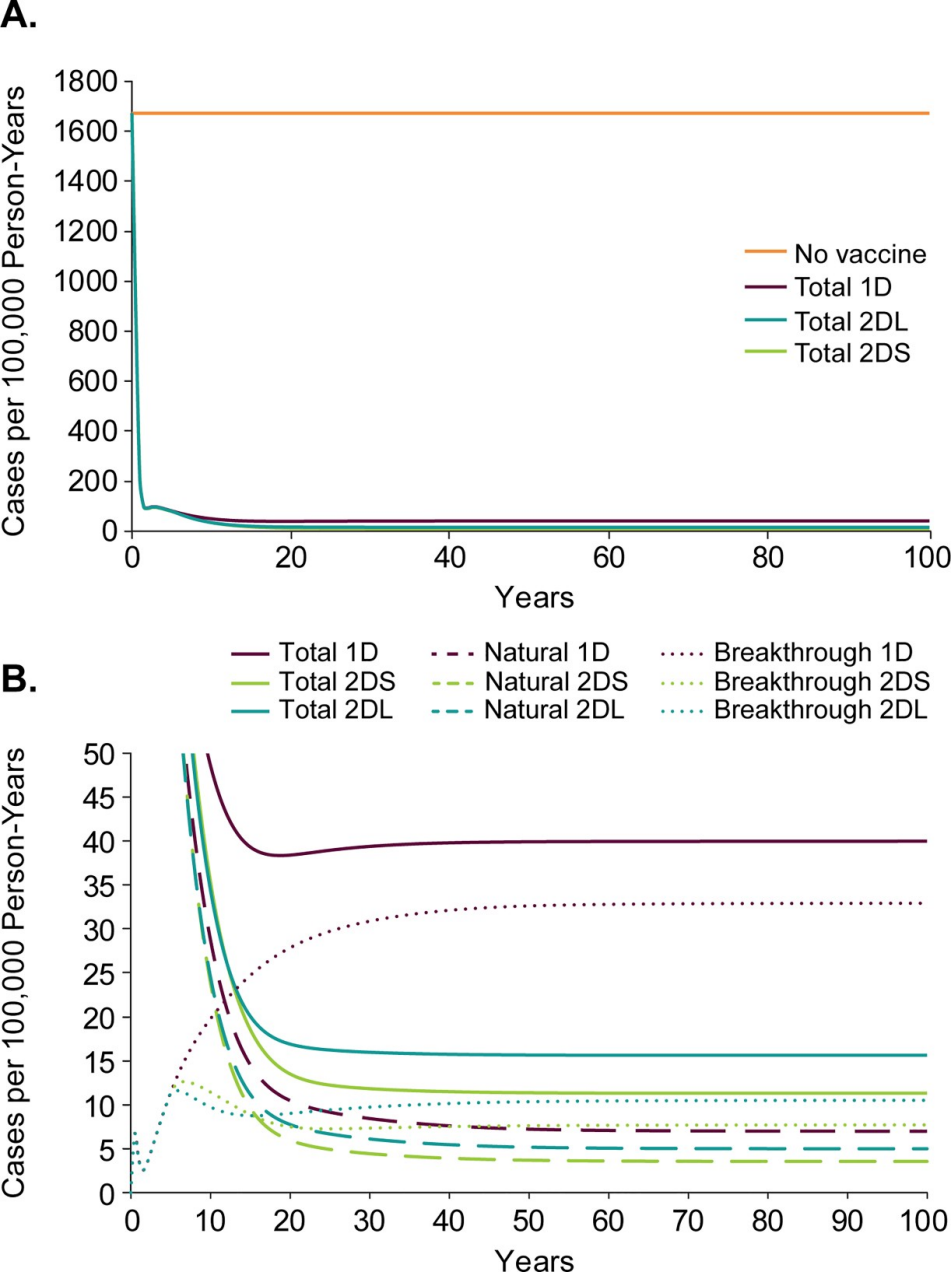
Varicella incidence in Turkey by varicella vaccination strategy. (A) over 100 years and (B) in greater detail over 100 years showing the natural (dashed) and breakthrough (dotted) contributions to the total varicella (solid) incidence for each of the strategies using the highly effective varicella vaccine. 1D, 1-dose; 2DS, 2-dose-short, and 2DL, 2-dose-long vaccination strategies.

The pre-vaccine era and no-vaccine strategy have a constant incidence of 1674 cases per 100,000 person-years. After introduction of the first varicella vaccine dose at 12 months of age (using the highly effective vaccine), the incidence rate falls to 80 cases per 100,000 person-years at 5 years, a 95% reduction in varicella disease (Fig 3). After the first 5 years the vaccination strategies diverge and, by 25 years, the incidence of varicella disease equilibrates at 39, 12, and 16 cases per 100,000 person-years for the 1-dose, 2-dose-short, and 2-dose-long strategies, respectively. The natural varicella component of the incidence is not substantially different for the three strategies—ranging from around 4 to 7 cases per 100,000 person-years—however, the rate of breakthrough varicella for the 1-dose strategy is 3 to 4 times larger than with either of the 2-dose strategies, which accounts for the differences in total incidence (Fig 3B).The cumulative varicella-related deaths, varicella cases, and congenital varicella cases that are avoided using each of the vaccination strategies over from 1 to 100 years are summarized in S1 Table.

The age distribution of varicella cases by vaccination strategy is depicted in S2 Fig over 100 years with exogenous boosting included in the model (the distribution is very similar with no exogenous boosting). With all strategies, <1% of all varicella cases are in the <1-year-old age group. Without vaccination, 58% of all varicella cases are in the 1- to 5-year-old age group, decreasing to 30%, 9%, and ≤3% for ages 5–10, 10–15, and ≥15 years. The introduction of any of the three vaccination strategies sharply reduces the proportion of cases in the 1- to 5-year-old age group and generally increases the proportion in the older age groups.

**Fig 4:**
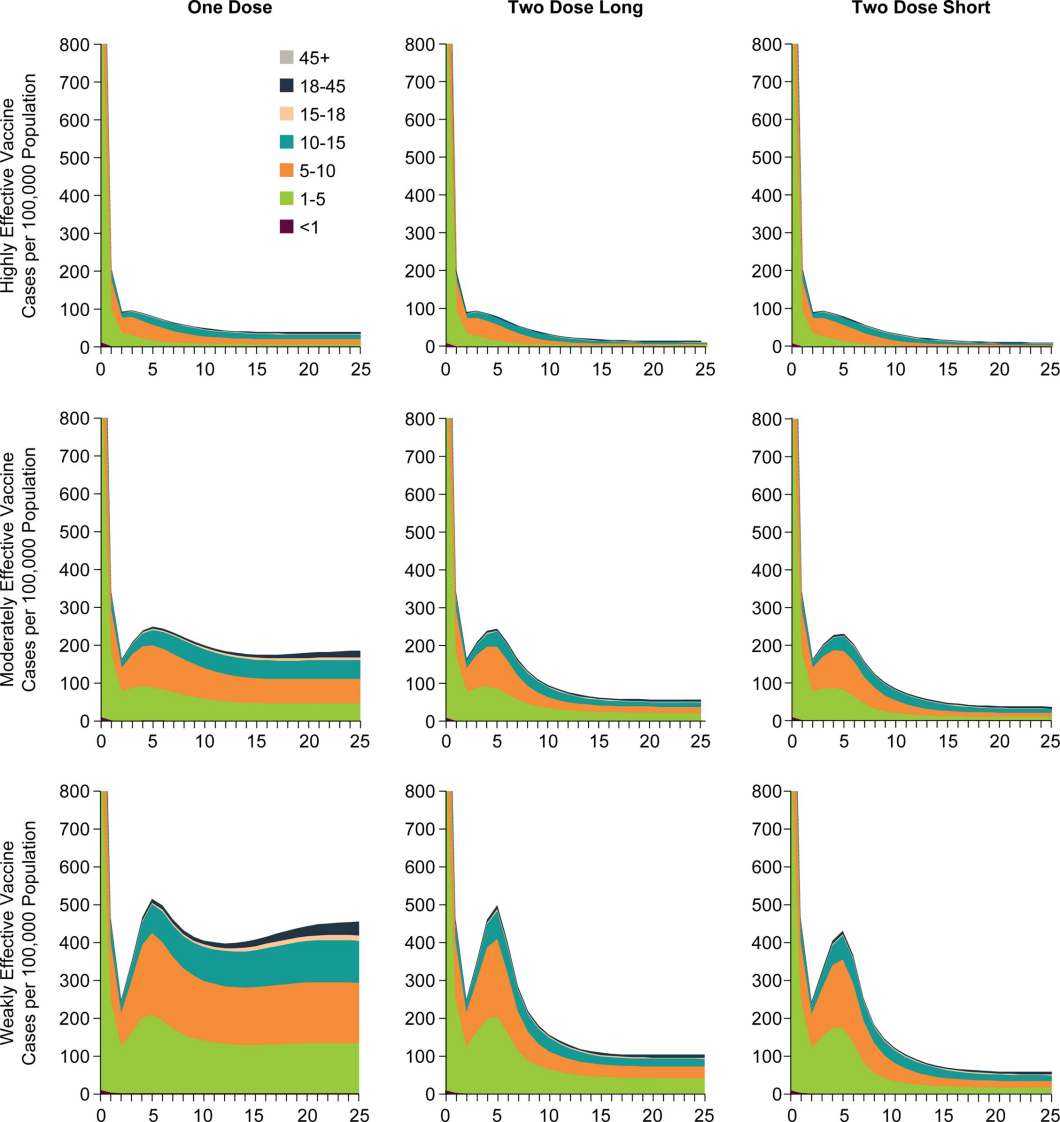
Age distribution of varicella cases over 25 years after start of varicella vaccination (years since start of varicella vaccination shown on x-axis). The plots depict three vaccination strategies (1-dose, 2-dose-short, and 2-dose-long) and three types of varicella vaccines of differing effectiveness (weakly, moderately, and highly effective).

We further investigated the age distribution of varicella cases using each of the three vaccine types (weakly, moderately, or highly effective) and the three vaccination strategies. The layered age incidence plots depict similar outcomes with each vaccine strategy for the highly effective vaccine and with both of the 2-dose strategies for the moderately effective vaccine (Fig 4). The results indicate that the two more effective vaccines can come close to eliminating varicella in children, with reductions in other age groups as well (S3 Fig). However, while the weakly effective vaccine produces overall short-term reductions in varicella disease, it has a bounceback effect a few years after initiation; and, of concern, the weakly effective vaccine may lead to an increase in varicella disease among ages 15–45 years (compared with the pre-vaccination period). This outcome results despite the use of optimistic assumptions in the model about the durability of that vaccine, which may be shorter in reality.

### Health and economic impact of highly vs. weakly effective vaccine

We considered whether the less effective but less expensive vaccine might be a cost-effective option in Turkey by comparing the three strategies for the weakly effective vaccine and the highly effective vaccine (i.e., 1-dose, 2-dose-short, and 2-dose-long), assuming a price of 55.36 TRY per dose of the weakly effective vaccine and 60.55 TRY per dose of the highly effective vaccine (see Table 1).

**Fig 5:**
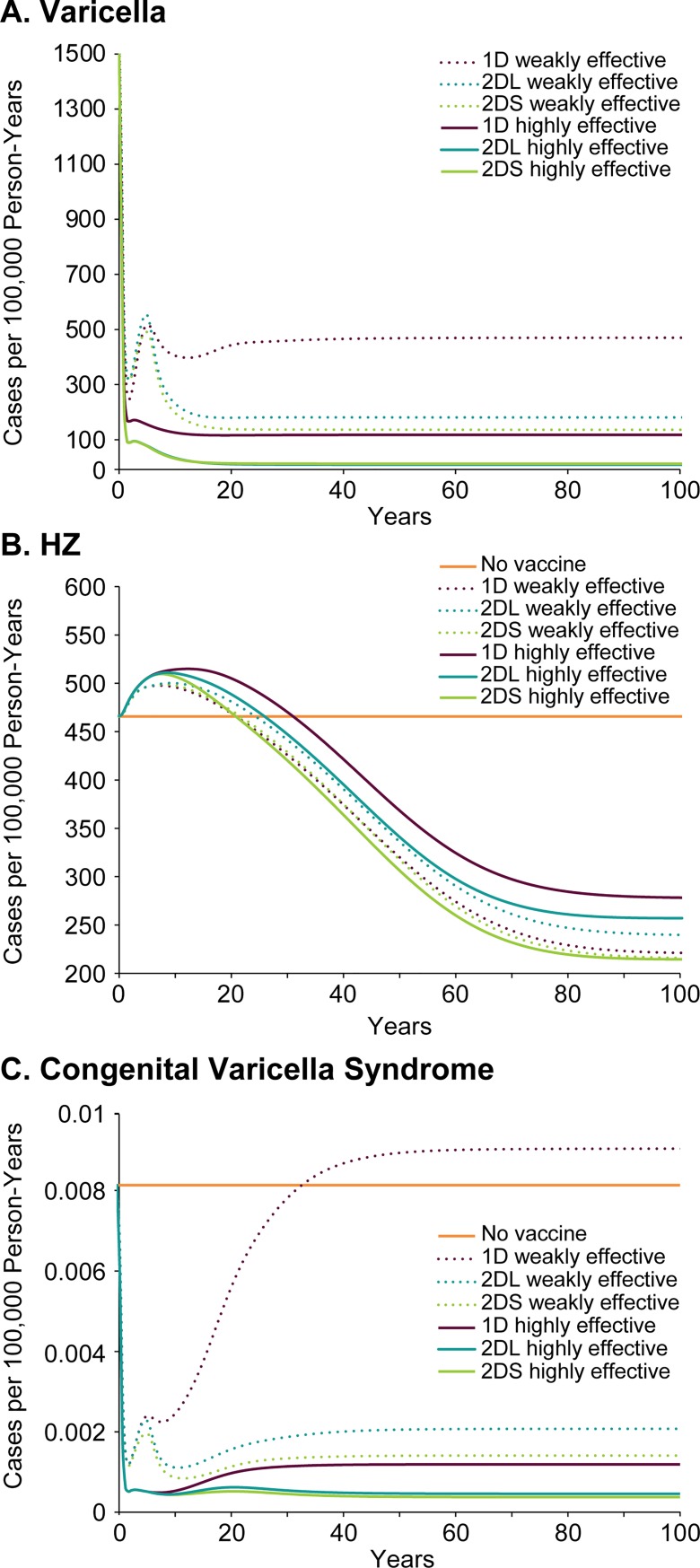
One- and 2-dose strategies with the weakly effective and the highly effective varicella vaccines: Predicted impact on incidence in the general population (per 100,000 person-years). (A) varicella, (B) herpes zoster, and (C) congenital varicella syndrome. (For varicella, the model is close to equilibrium by 25 years.) 1D, 1-dose; 2DS, 2-dose-short, and 2DL, 2-dose-long vaccination strategies.

The predicted varicella incidence for all strategies is that varicella would rapidly decline from the pre-vaccine era incidence of 1674 cases per 100,000 person-years. However, in strategies using the weakly effective vaccine, the incidence would rebound to around 50% of pre-vaccine levels within 5 years of starting the program (Fig 5A). Using two doses of the weakly effective vaccine close together might help prevent that rebound, but there are no data to support the hypothetical effectiveness of the second dose of the weakly effective vaccine; therefore, at most this possibility can be interpreted as a best-case scenario of the outcomes when using a 2-dose strategy with the weakly effective vaccine. Moreover, given the rapid waning of the first dose of the weakly effective vaccine, it seems unlikely that the second dose is as effective as we have portrayed in our model.

The impact on HZ incidence of the two vaccines and three strategies is more variable. The 2-dose programs using the highly effective vaccine have the least impact on HZ incidence, and the 2-dose-long strategy using the weakly effective vaccine causes the greatest and longest transient increases in HZ (Fig 5B).

Most striking, however, is the impact on the incidence of congenital varicella. While all programs using the highly effective vaccine decrease the incidence of congenital varicella, the 1-dose strategy using the weakly effective vaccine is predicted to cause a long-term increase in the rate of congenital varicella (Fig 5C). This finding is of concern because congenital varicella syndrome can lead to multi-system complications [56,57] and painful early onset of shingles [58,59].

When the projected distribution of direct and indirect costs by strategy and vaccine type is calculated over time, the lower cost of acquisition of the weakly effective vaccine is offset by higher projected medical costs (S4 Fig).

Table 2 shows the cost-effectiveness frontier analysis including all three strategies for both the weakly effective and highly effective vaccines for the scenario including exogenous boosting and including HZ costs. The no-vaccine and all strategies using the weakly effective vaccine are dominated by all of the strategies using the highly effective vaccine, i.e., they are more costly and less effective. The 1-dose highly effective vaccine strategy is the least costly, but the 2-dose-short highly effective vaccine strategy is cost-effective with an ICER of 38,023 TRY/QALY considering a willingness-to-pay (WTP) threshold of 55,597 TRY/QALY (based on the WHO recommended threshold of 1 times gross domestic product [GDP]). The 2-dose-long highly effective vaccine strategy is dominated by the 2-dose-short highly effective vaccine strategy (more costly and less effective). Other functions that achieved similarly good fits to the data did not change the dominance structure of the cost-effectiveness analysis.

**Table 2:** Cost-effectiveness frontier results at 100 years including "no vaccine" and the three vaccination strategies for each of the two vaccine types (weakly effective and highly effective): 1-dose (1D), 2-dose-short (2DS), and 2-dose-long (2DL) strategies for the scenarios including exogenous boosting effects and HZ costs and health outcomes.

Strategy	Total Costs^a^ (TRY)	Inc. QALYs^b^	ICER (TRY/QALY)
No vaccine	420.78	-0.01000	Dominated
1D-weakly effective	318.01	-0.00756	Dominated
2DS-weakly effective	323.18	-0.00726	Dominated
2DL-weakly effective	326.00	-0.00742	Dominated
1D-highly effective	310.50	-0.00749	-
2DS-highly effective	323.34	-0.00716	38,082
2DL-highly effective	326.19	-0.00734	Dominated

ICER, incremental cost-effectiveness ratio; Inc. QALYs, incremental quality-adjusted life-years; TRY, Turkish lira.

^a^Total costs are per capita and include direct and indirect costs.

^b^QALYs per capita.

### Impact on herpes zoster when using a highly effective vaccine

**Fig 6:**
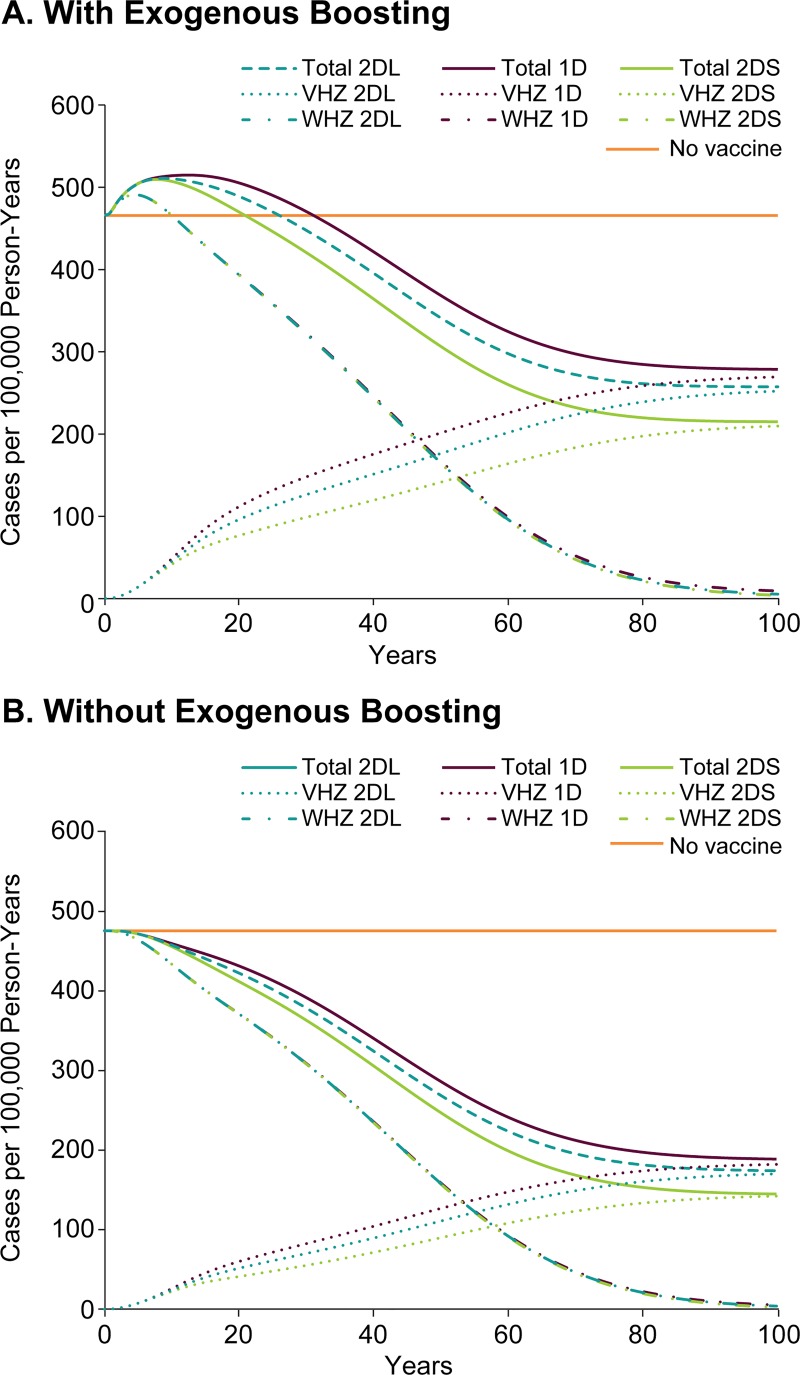
Predicted HZ incidence with no varicella vaccine or applying three different vaccination strategies using the highly effective varicella vaccine. (A) with exogenous boosting and (B) no exogenous boosting in the model. 1D, 1-dose; 2DS, 2-dose-short, and 2DL, 2-dose-long vaccination strategies; VHZ, vaccine-type HZ; WHZ, wild-type HZ.

The impact on HZ incidence of a varicella vaccination program using a highly effective vaccine (base case) varies according to whether exogenous boosting is included in the model. When including exogenous boosting, as reported by others [20,21,60], there is a notable increase in HZ incidence during the first 20 to 30 years of the vaccination program, after which time the HZ incidence eventually equilibrates at a lower rate than pre-vaccine (Fig 6A). The 1-dose varicella vaccination strategy leads to the longest period with increased HZ incidence (31 years) and the lowest long-term overall reduction in incidence (40%). The optimal long-term outcomes result from the 2-dose-short and 2-dose-long strategies, which lead to 54% and 45% reductions in incidence, respectively. In all cases wild-type HZ incidence begins falling below pre-vaccine levels by 10 years and is lower by >98% by 100 years, at which point nearly all of the HZ cases are vaccine-type HZ. At equilibrium the incidence rates of vaccine-type HZ are 270, 210, and 251 cases per 100,000 person-years for the 1-dose, 2-dose-short, and 2-dose-long strategies, respectively.

Considering the model without exogenous boosting effects, the most notable difference is that the HZ incidence never goes above the pre-vaccine incidence (Fig 6B). In addition, the incidence for each strategy after 100 years is reduced by 60%, 70%, and 63% for 1-dose, 2-dose-short, and 2-dose-long strategies, respectively, from the no-vaccine incidence. The incidence of wild-type HZ is reduced by approximately 99% for all three strategies while the vaccine-type HZ accounts for nearly all the HZ incidence by 100 years.

The age distribution of HZ cases by vaccination strategy is depicted with and without exogenous boosting in S5 Fig Over 100 years, the distribution of HZ cases for no vaccine peaks in the 15–30 and the 50–60 year old age groups at 19% and 16%, respectively, with other age groups ranging from 3% to 14% (S5A Fig). With the one-dose, two dose-short, and two dose-long strategies, the 0–15 year age group increases to 33%, 26%, and 31% of all varicella cases, respectively. While all age groups over 30 see a 3% to 8% reduction in cases in each age group and only a slight increase in the 15–30 year old age group. With exogenous boosting the age distribution of HZ cases is qualitatively similar to the distribution without exogenous boosting (S5B Fig).

### Costs and cost-effectiveness of highly effective vaccine when incorporating costs of HZ

The treatment-related and vaccine costs (direct, indirect, and total) for no vaccine and the 1-dose, 2-dose-short, and 2-dose-long strategies using highly effective vaccine are summarized in S2 Table according to four combinations of assumptions, i.e., with or without exogenous boosting and including or excluding HZ-related costs and benefits. As would be expected, the cumulative costs are substantially higher when HZ treatment is included than when HZ treatment is not included. The vaccine strategy costs are nearly the same for all four combinations except for some difference in the no-vaccine costs between with and without exogenous boosting scenarios because the presence of exogenous boosting does have a small effect on the varicella incidence.

When HZ costs are included, the inclusion in the model of exogenous boosting reduces discounted treatment costs by 35%, 39%, and 37% for 1-dose, 2-dose-short, and 2-dose-long strategies, respectively; and excluding exogenous boosting reduces discounted treatment costs by 44%, 46%, and 45%, respectively. When HZ costs are excluded, the inclusion in the model of exogenous boosting reduces discounted treatment costs by 96%, 97%, and 97% for 1-dose, 2-dose-short, and 2-dose-long strategies, respectively; and excluding exogenous boosting reduces discounted treatment costs by 96%, 97%, and 97%, respectively. The disparity between inclusion and exclusion of HZ costs arises because two-thirds of all varicella-related treatment costs are due to HZ and, while varicella vaccination reduces varicella incidence by nearly 98%, it reduces the HZ incidence by only about 20% to 50%, thereby leaving the largest contribution to the burden only partially mitigated.

**Fig 7:**
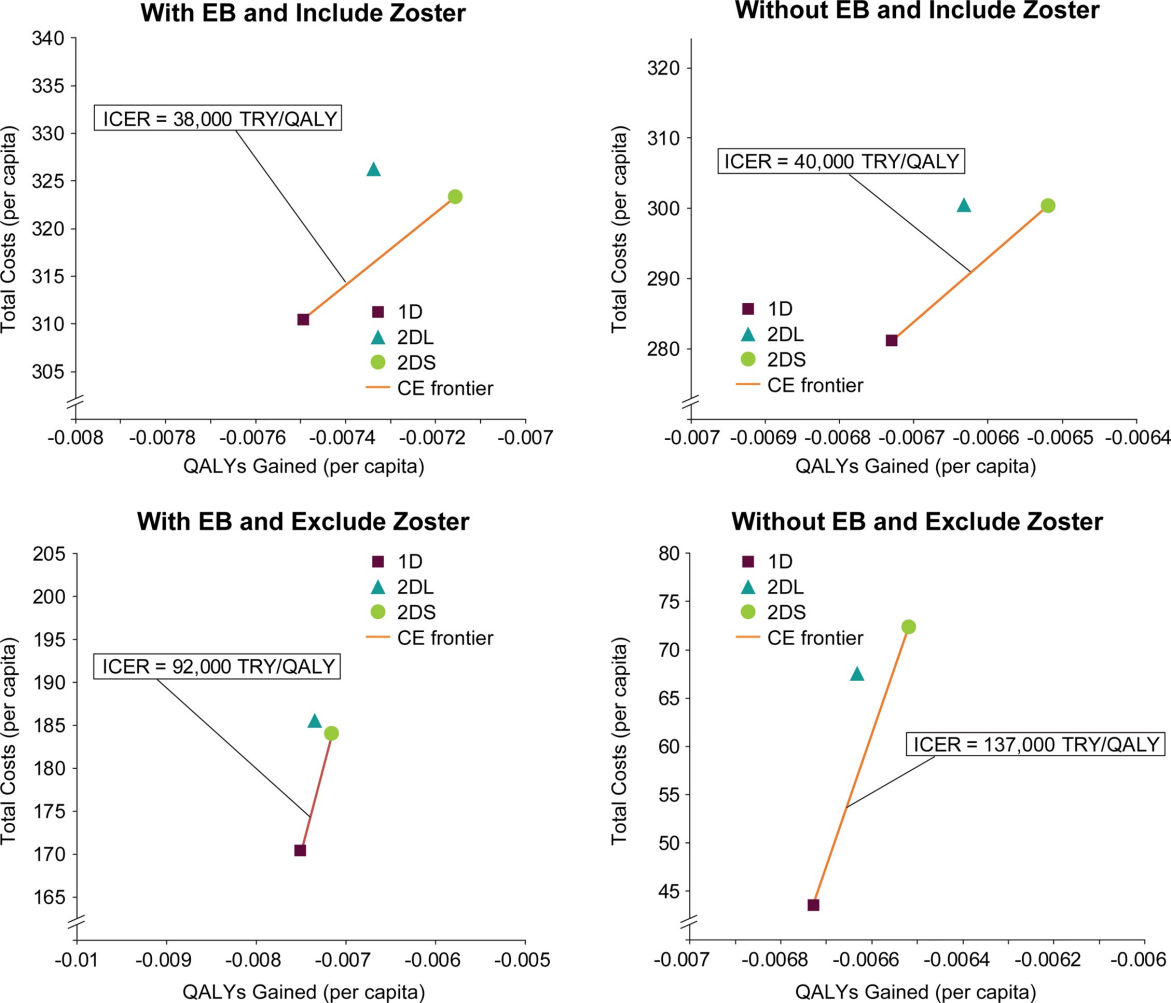
Cost-effectiveness plane for Turkey comparing varicella vaccination strategies with and without inclusion of exogenous boosting and/or HZ (zoster) in results, considering only the highly effective varicella vaccine. 1D, 1-dose; 2DS, 2-dose-short, and 2DL, 2-dose-long vaccination strategies; CE, cost-effectiveness; EB, exogenous boosting; ICER, incremental cost-effectiveness ratio; QALY, quality-adjusted life-years; TRY, Turkish lira.

With regard to cost-effectiveness of the four combinations of scenarios, with or without exogenous boosting the inclusion of HZ-related costs and health benefits leads to a substantially lower ICER for the 2-dose-short strategy (Fig 7). In particular, considering the WHO recommendation for willingness-to-pay (WTP) threshold of 1–3 times GDP of 19,618 USD = 55,597 TRY [38,39], the inclusion of exogenous boosting and HZ costs and health benefits leads to the 2-dose-short strategy being cost-effective although there is a temporary increase in HZ burden. This is in contrast to results from some other notable modeling studies using a similar modeling framework for exogenous boosting where the authors have concluded that universal childhood vaccinations programs may not be cost-effective due to the temporary increase in HZ burden [20,61]. Instead, when HZ costs and benefits are not included, the ICER for the 2-dose-short strategy is well beyond the WTP threshold. In all cases, the no-vaccine strategy (not shown in the plots) and the 2-dose-long strategies are dominated and the 1-dose strategy would be the most cost-effective.

### One-way sensitivity analysis results

**Fig 8:**
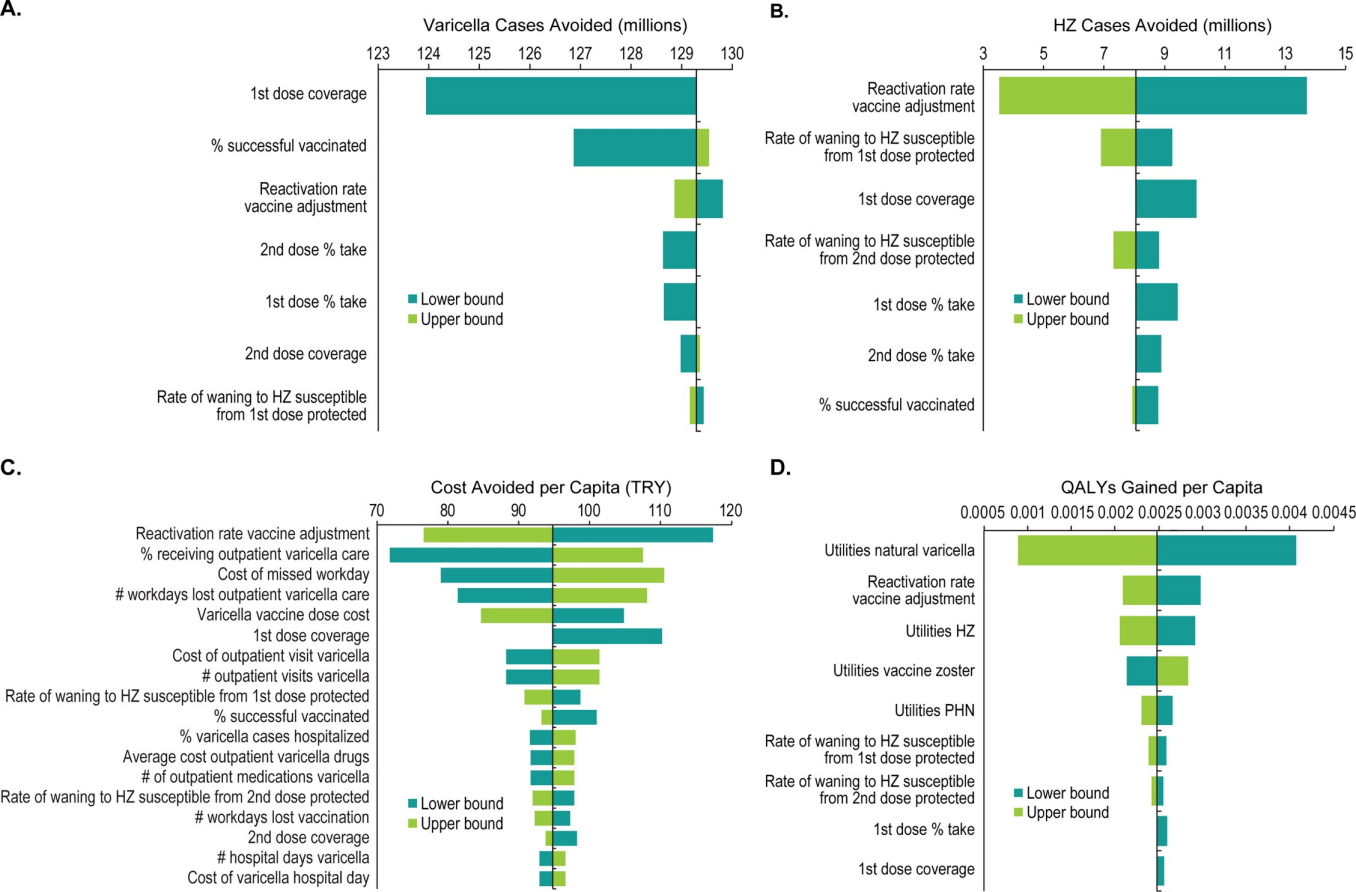
One-way sensitivity analysis: Tornado diagrams depicting parameter sensitivities in the comparison between no varicella vaccine vs. the 2-dose-short varicella vaccination strategy. (A) total cumulative varicella cases avoided after 100 years (showing parameters resulting in ≥3% range of change), (B) cumulative HZ cases avoided (showing parameters resulting in ≥0.1% range of change), (C) total cumulative discounted varicella costs avoided after 100 years (showing parameters resulting in ≥2% range of change), and (D) cumulative discounted QALYs gained after 100 years (showing parameters resulting in ≥2% range of change). HZ, herpes zoster; PHN, postherpetic neuralgia; QALY, quality-adjusted life-years; TRY, Turkish lira.

For the one-way sensitivity analysis we restricted ourselves to analysis of the total costs and QALYs of the 2-dose-short strategy relative to no vaccine and including exogenous boosting and HZ costs and benefits. The total number of varicella cases avoided after 100 years of the 2-dose-short program is most strongly influenced by first-dose coverage, followed by three vaccine parameters: overall percentage successfully vaccinated (vaccine take), and second- and first-dose vaccine take (Fig 8A). Similarly, the number of HZ cases prevented by the 2-dose-short program is most sensitive to the reactivation rate vaccine adjustment factor (χ is varied from 1/12 to ¼) (Fig 8B), leading to 56% more cases prevented and 70% fewer cases prevented respectively, both over 100 years. Effects of other parameters resulting in at least 0.1% range of changes are shown in Fig 8B.

For total costs, the parameter having the strongest influence is again reduction in risk of reactivation (Fig 8C), resulting in 19% increase and a 24% decrease in cost savings. The percentage of patients receiving outpatient care ranges from 72% to 100% and results in a decrease of 13% and an increase of 24% in savings, respectively.

The total of QALY losses prevented is most strongly influenced by the utility values for natural varicella (Fig 8D), which were varied from -20% to +20% of the base case value, leading to a range of -64% to +64% change in QALY losses prevented, respectively. The utility values for uncomplicated wild-type HZ were varied from -20% to +20% of base case values, leading to a range of -19% to +19% change in QALY losses prevented, respectively. Varying the reactivation rate vaccine adjustment factor from 1/12 to 1/4 results in a range of -20% to +16% change in QALY losses prevented, respectively.

### Probabilistic sensitivity analysis results

**Fig 9:**
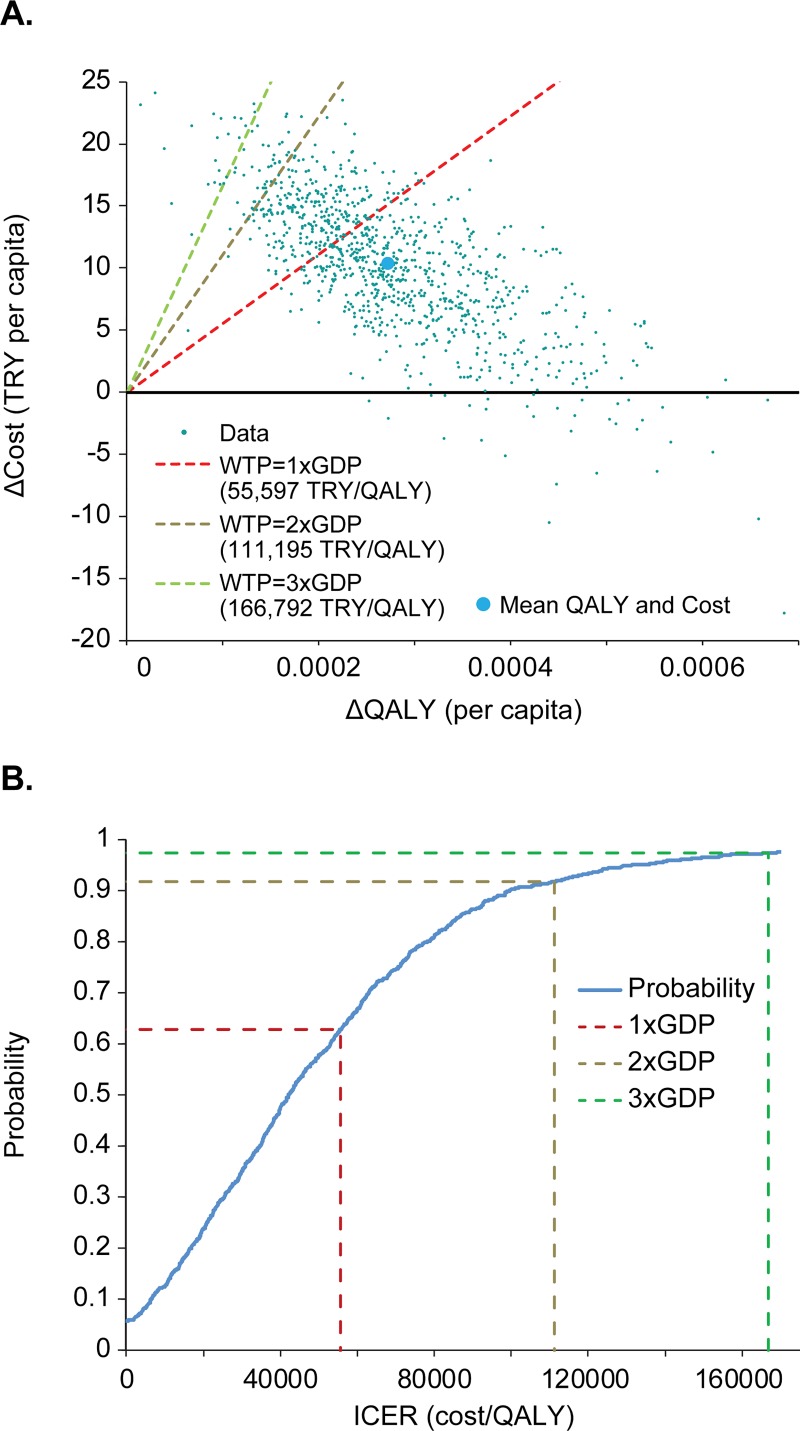
Results of the probabilistic sensitivity analysis. (A) probabilistic assessment against willingness-to-pay (WTP) thresholds and (B) cost-effectiveness acceptability curve for the 2-dose-short varicella vaccination strategy. GDP, gross domestic product; ICER, incremental cost-effectiveness ratio; QALY, quality-adjusted life-years; TRY, Turkish lira.

The probabilistic sensitivity analysis considered only the 1-dose and 2-dose-short programs with exogenous boosting and including HZ-related costs and outcomes. The resulting plot of incremental costs and QALYs is shown in Fig 9A. Considering a per-capita GDP of 55,597 TRY and using the WHO WTP thresholds of 1–3 times GDP per QALY (thus 55,597 TRY, 111,194 TRY, and 166,792 TRY per QALY, respectively), the 2-dose-short strategy is cost-saving as compared with the 1-dose program for 5.8% of the sample and cost-effective at 1–3 times GDP for 62.8%, 91.9%, and 97.5% of the sample, respectively (Fig 9B).

### Study strengths and limitations

The dynamic transmission model was designed to accommodate numerous, varying inputs of varicella vaccine effectiveness and aspects related to the natural history of varicella, of HZ, and of the interplay between varicella and HZ to compare health outcomes using different vaccination strategies. In addition, the model design supports detailed input of resource utilization, direct and indirect costs, and health utility values to estimate economic impact from payer and societal perspectives. This approach to cost-effectiveness analysis is aligned with recent consensus recommendations [62]. Moreover, the model was calibrated using Turkey-specific age distribution, fertility, and pre-vaccine varicella seroprevalence by age. Seroprevalence data rather than incidence data were used for the model calibration to eliminate the issue of underreporting of varicella cases, which is a common problem, as noted by Bechini et al. [63,64].

We provide estimates separately for natural and breakthrough varicella and HZ incidence over 50-year time horizons, as well as the age distribution of varicella cases over 25 years estimated for three levels of vaccine effectiveness and three vaccination schedules. Finally, we used both one-way and probabilistic sensitivity analyses to test the range of assumptions and conclusions.

Several limitations of this cost-effectiveness analysis should be considered when evaluating our findings. The model employed a static population age distribution and population size for Turkey, which could overestimate transmission in older people. Moreover, the population pyramid for Turkey is very monotonic/rectangular, and the size of most age groups is relatively constant until age 60, thus through the ages most likely to be impacted by exogenous boosting. Therefore, we believe the age distribution used is a reasonable approximation to a realistic age-specific social contact matrix in this setting.

We acknowledge that demographic changes not captured by the model, such as changes in fertility trends in Turkey, could potentially impact the health outcomes of using different vaccination strategies [25,65]. The importance of demographic changes over time is particularly pertinent for childhood diseases. For example, declining birth rates in Italy were associated with a decline in measles incidence even before the institution of a national measles immunization program [66]. Similarly, large changes in social contact patterns, such as the increase in day-care programs and enrolment of preschool children in France, can alter the incidence patterns of childhood diseases [67].

Another study limitation is the use of surrogate data for HZ from Israel. A review of HZ incidence reports considerable variability among geographical settings, including among sociodemographically and economically similar countries, such as Germany, the Netherlands, and Belgium, with reported differences not only in the average incidence but also in the shape of the age-specific curve in older ages [68]. However, both Turkey and Israel show similar distribution of the population across age groups (except for Israel having longer life expectancy), and the limited data available on VZV seropositivity in Turkey for ages ≥15 years indicated a reasonably good match between Turkey and Israel [35]. Therefore, given the limited availability of HZ data for Turkey at the time we did the analysis, the proxy of using Israeli data seemed reasonable. Further support for our decision is provided by the results of a recently published seroprevalence study of one province in Turkey (published after our analysis had been completed) that are broadly similar to the Israeli data, particularly given the age groups covered [48]. Nonetheless, we acknowledge this study limitation.

In addition, we made the assumption that breakthrough varicella infection is characterized by both a reduced infectious period and lower infectivity. While this assumption is supported by recent work, including the study of Cheng et al. [12], there remains the potential for having underestimated the relative contribution of breakthrough varicella infections.

Finally, the appropriate approach to modeling exogenous boosting effects is still being actively debated, and the currently modeled mechanism may not be realistic [5,24]. We have used the temporary full immunity model of exogenous boosting, as have others [20,61]. However, while even the existence of the exogenous boosting effect is still being questioned [69], there is also ongoing research on alternative modeling of the exogenous boosting mechanism [5]. Different modeling approaches have led to very different epidemiologic results. For example, Guzzetta et al. [3] have shown that the progressive immunity model increases both the duration and magnitude of the increase in HZ incidence. This would likely result in substantial increased HZ burden and decrease in the cost-effectiveness of the varicella program relative to the temporary full immunity assumption.

## Conclusions

We developed a dynamic transmission model to accommodate varying assumptions of varicella vaccine effectiveness, vaccination schedules, and costs, as well as effects of exogenous boosting and HZ-related costs, to explore vaccination strategies for Turkey. With regard to health impact, the 2-dose-short strategy of UVV at 12 and 18 months of age results in greater reductions in varicella incidence rate in Turkey, equilibrating at 25 years, than the 1-dose and 2-dose-long strategies. When we further considered the exogenous boosting and HZ-related cost scenarios in the model, we found that with or without exogenous boosting, the inclusion of HZ-related costs and health benefits in the model resulted in a substantially lower ICER for the 2-dose-short strategy than the other strategies, although with exogenous boosting there is a temporary increase in HZ burden during the first 20 to 30 years of the vaccination program, after which time the HZ incidence eventually equilibrates at a lower rate than pre-vaccine. The results of one-way and probabilistic sensitivity analyses indicate that these results are sensitive to parameters of vaccine effectiveness, including vaccine take and duration of immunity. Overall, our findings support the cost-effectiveness of adding a second varicella vaccination at 18 months of age to the UVV schedule in Turkey. One limitation to this conclusion is that we do not consider the possible cost-effectiveness of a joint varicella and herpes zoster vaccination program, which could lead to different conclusions.

This dynamic transmission model can be applied to other settings, using population-specific parameters, to evaluate health effects and cost-effectiveness of varicella vaccination strategies, with or without the inclusion of HZ vaccination as well.

## Supporting information

S1 Appendix Detailed description of the model. (PDF)

S2 Appendix Turkey-specific data used in the model. (PDF)

S1 Fig Model calibration: Modeled HZ incidence rate and the adjusted observed incidence rate. (PDF)

S2 Fig Age distribution of varicella cases by vaccination strategy: Percentage of total varicella incidence at 100 years. (PDF)

S3 Fig Age-specific reductions in varicella incidence at 5, 10, and 25 years after start of vaccination using three vaccination strategies and three types of vaccines of differing effectiveness. (PDF)

S4 Fig Distribution of costs over time by varicella vaccine type and vaccination strategy. (PDF)

S5 Fig Age distribution of HZ cases by vaccination strategy. (A) with exogenous boosting and (B) without exogenous boosting: percentage of total HZ incidence at 100 years.
(PDF)

S1 Table Cumulative results for varicella disease in Turkey using 1-dose (1D), 2-dose-short (2DS), or 2-dose-long (2DL) varicella vaccination strategy over 1–100 years. (PDF)

S2 Table Direct, indirect, and total costs for Turkey using one-dose (1D), 2-dose-short (2DS), or 2-dose-long (2DL) varicella vaccination strategies with a highly effective vaccine, with and without including exogenous boosting and with and without including HZ-related costs. (PDF)
