# Supplementary material for: Cost-effectiveness analysis of universal varicella vaccination in Turkey using a dynamic transmission model
Source: PLoS One. 2019 Aug 13;14(8):e0220921. doi: 10.1371/journal.pone.0220921 (PMC6692038; doi:10.1371/journal.pone.0220921)
Supplement: S1 Appendix — (PDF) [file pone.0220921.s001.pdf]

S1 Appendix. Detailed description of the model

Cost-effectiveness analysis of universal varicella vaccination in Turkey using a dynamic transmission model

1. Supplementary Methods

1.1. Demographic model

The population is divided into N age groups defined by the age intervals $[a_{i-1}, a_i]$, where $a_1 < a_2 < \ldots < a_n = \infty$. The number of individuals $n_i(t)$ at time t in the age interval $[a_{i-1}, a_i]$ is the integral of the age distribution function from a_{i-1} to a_i. Assuming that the population distribution has reached a steady state with zero growth or decay, Hethcote [1] derived a system of N ordinary differential equations (ODEs) for the sizes of the N age groups. The simple demographic model divides the population into discrete age groups defined to evaluate outcomes and to support typical varicella vaccination schedules for the first and second varicella doses and to allow for HZ vaccine scheduling (Table A).

New additions to the population enter at rate Λ. Persons are transferred between successive age groups at an age-specific per capita rate d_i per year. The transfer rate d_i is given by the following (with $d_N = 0$):

$$d_i = \frac{\mu_i}{e^{(a_i - a_{i-1})\mu_i} - 1}$$

The population distribution is described by a set of ordinary differential equations describing the time dependence of the populations within each of the age ranges,

$$\frac{dn_1}{dt} = \Lambda - (d_1 + \mu_1)N_{1,}$$

$$\frac{dn_i}{dt} = d_{n-1}n_{i-1} - (d_i + \mu_i)n_i,$$

the assumption of stationary population structure and size give the equilibrium relations $\Lambda = (\mu_1 + \ d_1)n_1^* \ and \ d_{i-1}n_{i-1}^* \ (\mu_i + \ d_i)n_i^*$.

So the age group population necessary to maintain a constant population distribution is

$$n_i(0) = n_i^* = \Lambda \prod_{j=0}^{i} \frac{d_{j-1}}{d_j + \mu_j}$$

With $d_0 = 1$. And the population size necessary to balance the given mortality is

$$\Lambda = (\mu_1 + d_1)\left(1 + \sum_{i=2}^{ages} \prod_{j=2}^{i} \frac{d_{j-1}}{d_j + \mu_j}\right)^{-1}$$

Here, n_i^* is the equilibrium population in age group i, hereafter simply referred to by n_i.

We define a 1-month period for neonatal specific outcomes. Beginning at 11 months we define 1-month age groups up to 24 months old to support typical first varicella vaccine dose scheduling. From 2 years old through 20 years old we define 1-year age groups to support second varicella dose strategies that are typically defined by year of age. Beyond 20 years we define 5- and 10-year age groups allowing for herpes zoster (HZ) vaccine scheduling and sufficiently detailed outcomes by age group (Table A).

Table A. Model age groups.

Life stage	Age groups in months (m) or years (y)[a]					
Neonate	0 – 1m					
Infant	1m – 6m	6m – 11m	11m – 12m			
	12m – 13m	13m – 14m	14m – 15m	15m – 16m	16m – 17m	17m – 18m
	18m – 19m	19m – 20m	20m – 21m	21m – 22m	22m – 23m	23m – 24m
Young child	2y -3y	3y – 4y	4y – 5y	5y – 6y		
Child	6y – 7y	7y – 8y	8y – 9y	9y – 10y	10y – 11y	11y – 12y
Adolescent	12y – 13y	13y – 14y	14y – 15y	15y – 16y	16y – 17y	17y – 18y
Young adult	18y – 19y	19y – 20y	20y – 25y	25y – 30y	30y – 35y	
Middle-aged adult	35y – 40y	40y – 45y	45y – 50y	50y – 55y		
Older adult	55y – 60y	60y -65y	65y – 70y	70y – 80y	80y – 90y	90y +

[a]Age group boundaries are closed on the left (boundary is included in the group) and open on the right (boundary is not included in the group).

1.2 Epidemiologic model

Below we describe the model in four sections with corresponding diagrams. The definitions used to define each compartment of the model are described in Table B, and the base model parameters are summarized in Table C.

Table B. Varicella-zoster model compartment labels and definitions.

Compartment label	Description
**m**	Population with maternal immunity
**s**	Susceptible to natural varicella infection
**s^1**	Susceptible to natural varicella infection after failed 1st dose of varicella vaccine
**s^2**	Susceptible to natural varicella infection after failed 2nd dose of varicella vaccine
**Natural varicella – wild herpes zoster (HZ)**	
**e**	Exposed to varicella infection
**e^f**	Exposed to varicella infection after failed 1st or 2nd dose of varicella vaccine
**i**	Infectious varicella
**r**	Recovered from varicella (high immunity to HZ)
**rc**	Recovered from congenital varicella (high immunity to HZ)
**w**	Waned zoster immunity (low immunity to HZ)
**z**	Wild zoster infected
**rz**	Wild Zoster recovered
**vzw**	HZ vaccinated protected
**wz**	HZ vaccinated susceptible
**Varicella vaccination**	
**v1**	1-dose varicella vaccination protected
**sv1**	Susceptible to varicella after 1st dose of vaccine
**$v1^1$**	2nd dose varicella vaccinated after failed 1st dose (biological behavior is like first dose)
**$sv1^1$**	Varicella protected by 2nd dose varicella vaccination after failed 1st dose
**v2**	2-dose varicella vaccination protected
**sv2**	Susceptible to varicella after 2nd dose of vaccine
**Breakthrough varicella – wild herpes zoster (HZ)**	
**evb**	Exposed to varicella infection after vaccination (breakthrough varicella)
**ivb**	Infectious breakthrough varicella
**rvb**	Recovered from breakthrough varicella (high zoster immunity)
**wvb**	Waned zoster immunity (low zoster immunity)
**zwild**	Wild zoster after breakthrough varicella
**vzb**	HZ vaccinated protected after breakthrough varicella
**wvbz**	HZ vaccinated susceptible after breakthrough varicella
**Varicella vaccinated – vaccine herpes zoster (HZ)**	
**rvv**	Permanent varicella and high HZ immunity after vaccination
**wvv**	Low HZ immunity after varicella vaccination
**zvacc**	Zoster infection after varicella vaccination
**vzv**	HZ vaccinated protected after varicella vaccination
**wvvz**	HZ vaccinated susceptible after varicella vaccination

Table C. Base model parameters.

Symbol	Description	Value	Source
Disease-specific parameters			
$1/\omega_m$	Average period of passive immunity	6 months	[2]
$1/\epsilon_n$, $1/\epsilon_{vb}$	Average latent period (natural, breakthrough varicella)	14 days	[3]
$1/\gamma_n$	Average infectious period for natural varicella	7 days	[4]
$1/\gamma_{bv}$	Average infectious period for breakthrough varicella	4.5 days	[4]
$1/\eta_n, 1/\eta_{vv}, 1/\eta_{vb}$	Average length of HZ case (wild-type, vaccine-type, wild-type after breakthrough varicella)	28 days	[5]
$1/\delta_n$, $1/\delta_{vv}$, $1/\delta_{vb}$	Average duration of high HZ immunity	79.7 years	[6]
ρ_v	Relative infectivity of breakthrough varicella infection	50%	[7]
ρ_z	Relative infectivity of HZ infection	0.07	[8]
$\zeta_n, \zeta_{vv}, \zeta_{vb}$	Percentage of contacts leading to exogenous boosting	100%	[9]
$\xi_n, \xi_{vv}, \xi_{vb}$	Rate of endogenous boosting	0	Assumed
χ	Reactivation rate factor on vaccine arms	1/6	[6]
$\sigma(a)$	HZ reactivation rates	see S2 Appendix, Table D	From calibration
β	Transmission matrix	see S1 Appendix text	From calibration
μ	Mortality	see S1 Appendix text	Derived
Fertility	Births by age of mother	Births/1000	[10]
	Age (years)		
	15 - 20	31.626	
	20 -25	114.24	
	25 – 30	136.668	
	30 - 35	85.386	
	35 – 40	39.69	
	40 – 45	10.5	
	45 - 50	1.89	
cfr_z	HZ case fatality ratio by age	cases / 100,000	[11]
	<5	0	
	5 - 15	1	
	15 – 80	2	
	80+	61	
cfr_n, cfr_{vb}	Varicella case fatality ratio	see S1 Appendix text & S2 Appendix, Table C	[12,13]
α_c	Risk of congenital varicella	0.4%	[14]

Symbol	Description	Value	Source
p_{phn}	Age-specific probability of HZ developing into PHN	%	[15]
	<5	0	
	5 - 15	1	
	15 – 50	4	
	50 - 70	11	
	70+	31	
d_{phn}	Average duration of PHN (days)	511	[15]
Varicella vaccine-related parameters (default vaccine, Varivax®)			
F=(1-P)	Vaccine failure	4%	[16]
T_1	Varicella vaccine 1st dose take	100%	[16]
T_2	Varicella vaccine 2nd dose take	100%	[16]
$1/\omega_{v1}$	Average duration of 1st dose varicella vaccine immunity to varicella	25 years	[16]
$1/\omega_{v2}$	Average duration of 2nd dose varicella vaccine immunity to varicella	77 years	[16]
Varicella vaccine-related HZ protection parameters (assumed same for all varicella vaccines)			
$1/\pi_1$, $1/\pi_2$,	Average duration of 1st, 2nd dose varicella vaccine induced high HZ immunity	same as vaccine duration of protection	Assumed
$1- b_{v1}$, $1- b_{v2}$	Varicella vaccine 1st, 2nd dose degree of protection	0	Assumed
k_{v1}, k_{v2}	Percentage of 1st /2nd dose protected who are boosted to full HZ protection from contact with varicella	100%	[16]
Zoster vaccine-related parameters (based on Zostavax®)			
$1 - b_z$	HZ vaccine degree of protection[a]	69.9%	[17]
$1/\omega_z$	Average duration of HZ vaccine immunity	4.3 years	[17]

HZ, herpes zoster; PHN, postherpetic neuralgia; VZV, varicella-zoster virus.

[a]Degree of protection and duration of protection are derived from Tseng et al. [17] assuming simple exponential decay and are specific to Zostavax® (Merck & Co., Inc.).

1.2.1 Force of infection and mixing matrix

The force of infection (FOI) for age group k is the sum of all contacts with infectives in other age groups, j. Let ρ_v, and ρ_z be the relative infectiousness of people infected with breakthrough varicella and HZ, respectively, compared with people infected with varicella.

$$\lambda_k(t) = \sum_{j=1}^{ages} \beta_{k,j}[i_j(t) + \rho_v ivb_j(t) + \rho_z \left(z_j(t) + zwild_j(t) + zvacc_j(t)\right)] \qquad (1)$$

Here, $\beta_{k,j}$ is the mixing matrix which is the disease transmission rate to people in age group k from people in age group j. The mixing matrix is population specific and is determined during calibration.

1.2.2. Vaccination models

The model provides options to implement both varicella vaccination and HZ vaccination and supports one regimen of vaccination for each vaccine: for example, an individual can receive no more than two doses of varicella vaccine and no more than one dose of HZ vaccine.

Varicella vaccination. The varicella vaccination model implements a very similar structure to that of van Hoek et al. [16]. The vaccine may fail completely for a proportion (1-P) of the vaccinees, and these individuals are biologically equivalent to those who never received the vaccine. Of the fraction (P) for whom the vaccine does not fail, a proportion T_1 will become fully protected from varicella and 1-T_1 will become partially protected. Those fully protected experience the protection waning at a rate ω_1 and become partially protected. Partially protected individuals have a degree of protection (1-b_v) from varicella. The second dose of varicella vaccine follows a similar pattern except it is usually assumed that second-dose failure rates are 0.

The vaccine parameters chosen for the model are derived by van Hoek et al. [16] using published data from a 10-year follow up study of Varivax for both one and two doses (see Table C). It is important to understand that the values and meaning of the parameters P, T, and b_v are dependent on the model structure. In particular, Brisson et al. [9,18] define the transitions slightly differently in their varicella vaccine model: while they include the notion of vaccine failure (F in their model, (1-P) in ours), the proportion of persons who acquire full protection is T (in our model is PT) and those becoming partially protected is $1 - \mathrm{F} - \mathrm{T}$. Thus, the meaning of "T" is different in each of these models.

Herpes zoster vaccination. The HZ vaccination model implements the "take/degree/waning" model described by McLean and Blower [19]. In this model the HZ vaccine provides a degree of protection (1- b_z) to a proportion, T_z, of vaccinees and the rest, (1- T_z), are fully susceptible. The full protection is subject to a waning rate ω_z.

Births and natural varicella. Fig A illustrates the model flow from birth through natural varicella and wild zoster.

People are born at a constant birth rate, Λ. People are born into the congenital varicella recovered compartment (rc) at a rate

$$\Lambda_c(t) = \Lambda\, \alpha_c \sum_{a=1}^{ages} f_a \left(i_a(t) + ivb_a(t)\right) \quad (2)$$

With f_a the fraction of women giving birth in age group a, $\left(i_a(t) + ivb_a(t)\right)$ is the number of people in age group a that are infected at time t, and α_c is the proportion of infected mothers that transmit varicella to their fetus. We have excluded activated HZ infection from the congenital calculation since there is no evidence that varicella virus is transmitted to the fetus during an HZ infection [20,21].

People are also born into the susceptible compartment (s) at a rate

$$\Lambda_s(t) = \Lambda \sum_{a=1}^{ages} f_a \left(s_a(t) + s_a^1(t) + s_a^2(t) + sv1_a(t) + sv1_a^1(t) + +sv2_a(t)\right) \quad (3)$$

The remaining births are born into the maternally immune compartment (m) at a rate $\Lambda - \Lambda_c(t) - \Lambda_s\,(t)$. Maternal immune individuals (m) become susceptible to varicella infection (s) at a constant, age independent rate, ω_m.

Susceptible individuals (s, s^1, s^2) acquire latent natural varicella infection (e, e^f) at an age and time dependent rate $\lambda_k(t)$, the FOI (Equation (1)). Latently infected individuals become infectious (i) at a constant, age-independent rate ε_n. Infectious individuals clear varicella infection and become permanently immune to varicella and acquire high immunity to HZ (r) at a constant, age-independent rate γ_n or die at a constant, age dependent rate $\text{cfr}_n(a)\gamma_n$, where cfr is the case fatality risk (or case fatality ratio). High HZ immunity (r, rc) wanes to HZ susceptible (w) at a constant, age-independent rate δ_n.

HZ-susceptible individuals (w) may either

- acquire wild-type HZ (z) at an age-dependent rate $\sigma(a)$ or
- be boosted back to high zoster immunity (r) through exogenous boosting at an age and time dependent rate $\zeta_n(a)\lambda_a(t)$ (exogenous boosting) and a constant, age-dependent rate $\xi_n(a)$ (endogenous boosting).

High immunity (r, rc) and HZ-susceptible (w) individuals may be vaccinated with zoster vaccine and

- acquire a degree of protection, $(1 - b_z)$, from HZ reactivation (vzw) in proportion $T_z\, \theta_z$(a) to the aging rate or

- become susceptible to HZ reactivation (wz) in proportion $(1 - T_z)\ \theta_z$(a) to the aging rate.

Those who are partially protected from HZ reactivation (vzw) may acquire HZ at an age dependent rate $b_z\sigma_n(a)$ or may become fully susceptible to HZ (wz) at a constant, age-independent rate ω_z. The fully susceptible individuals (wz) may acquire wild-type HZ (z) at an age-dependent rate $\sigma(a)$. Wild-type HZ infected individuals (z) recover (rz) at constant rate η_n or die at a rate $\text{cfr}_z(a)\eta_n$ (Fig A).

Fig A. Partial model diagram detailing susceptible and maternally immune through natural varicella and wild zoster compartments and transitions. (see Table B for compartment labels and definitions.)

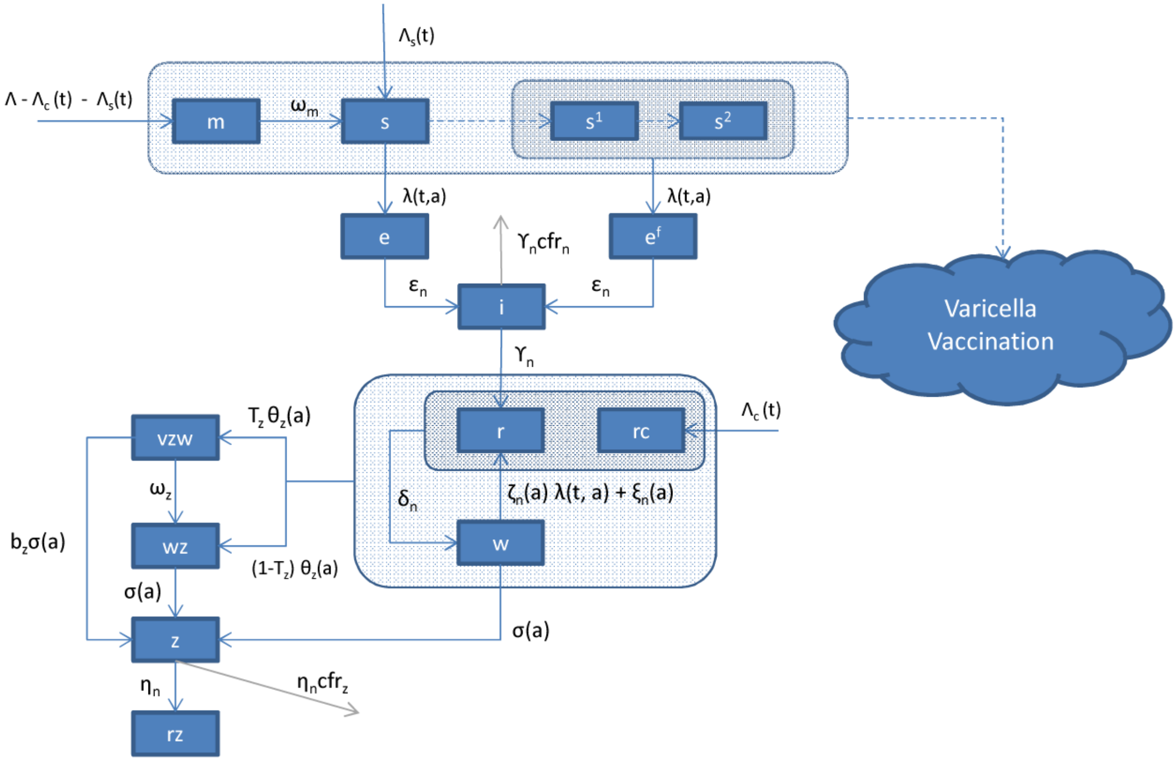

1.2.3. Varicella vaccination

The varicella vaccination model (Fig B) allows for two doses of varicella vaccine whereby each dose may either fail completely to provide any protection leaving individuals susceptible to natural varicella (s^1, s^2); provide full temporary immunity to varicella (v1, v2); or leave individuals susceptible to breakthrough (less severe) varicella infection. We have included the compartments s^1 and s^2 to allow the inclusion of vaccination history for those who fail to seroconvert. These cases can then be considered as breakthrough varicella from a clinical accounting perspective (counted as breakthrough cases since they receive vaccine), but treated as natural varicella from a biological perspective (since they contribute to the force of infection like natural varicella).

The vaccine transitions are proportional to the rate of aging into the varicella vaccination target age group from the previous age group [22]. We allow for flexible varicella vaccination schedules including catch-up programs for 1-year and multiple age groups; multiple years for one age group; and first dose on second-dose schedule for multiple years. The age-dependent proportionality constant representing the fraction of the target age group receiving one or two doses of varicella vaccine is given by,

$$\Theta_1(t,a) = \begin{cases} \theta_1, & a = a_1 \\ \theta_c, & a \in \{c_1, .., c_n\} \wedge t < \tau_c \\ \theta_s, & a = a_2 \ \wedge t < \tau_s \\ 0, & \text{otherwise} \end{cases} \quad (4$$

$$\Theta_2(a) = \begin{cases} \theta_2, & a = a_2 \\ 0, & \text{otherwise} \end{cases} \quad (5$$

Where a_1 is the age receiving the first dose of vaccine; the set $\{c_1, .., c_n\}$ are ages at which a catch-up vaccine is administered and τ_c is the duration of the catch-up campaign; $a_2$is the age receiving the second dose of vaccine and θ_s is the proportion receiving the first dose on the second-dose schedule where τ_s is the duration of the second opportunity catch-up program.

Maternally immune (m) and varicella susceptible individuals (s) who receive one dose of varicella

- transition to first-dose vaccine protected (v1) in proportion $(1-P)\ T_1\Theta_1(t,a)$ to the aging rate,
- transition to varicella susceptible after first dose (sv1) in proportion $(1-P\ )(1-T_1)\ \Theta_1(t,a)$ to the aging rate, or
- transition to susceptible after failed first dose (s^1) in proportion $P\ \ T_1\ \Theta_1(t,a)$ to the aging rate

where P is the proportion of vaccinees who "fail" to seroconvert and are biologically equivalent to unvaccinated and T_1 is the first dose "take."

Individuals with first-dose protection (v1) can

- become susceptible to varicella breakthrough infection (sv1) at a constant, age-independent waning rate ω_{v1}
- transition to second-dose vaccine protected (v2) in proportion $T_2\Theta_2(a)$ to the aging rate or
- transition to varicella susceptible after second dose (sv2) in proportion $(1 - T_2)\ \Theta_2(a)$ to the aging rate.

Individuals susceptible to varicella after the first dose (sv1) can

- transition to second-dose vaccine protected (v2) in proportion $T_2\Theta_2(a)$ to the aging rate or
- transition to varicella susceptible after second dose (sv2) in proportion $(1 - T_2)\ \Theta_2(a)$ to the aging rate.

When the first dose of varicella vaccine fails, the vaccinees may then go on to receive the second dose on the second-dose schedule, incurring second-dose costs but biologically behaving as though they have received only one dose. These individuals, susceptible to varicella after first-dose failure (s^1), can

- transition to 1st dose vaccine protected after 1st dose failure ($v1^1$) in proportion $(1 - P)\ T_1\ \Theta_2(a)$ to the aging rate
- transition to susceptible after 1st dose protected after 1st dose failure ($sv1^1$) in proportion transition to 1st dose vaccine protected after 1st dose failure ($v1^1$) in proportion $(1 - P)\ (1 - T_1)\ \Theta_2(a)$ to the aging rate
- transition to susceptible after 2nd dose failure (s^2) in proportion $P\ \Theta_2(a)$ to the aging rate

Fig B. Varicella vaccination: partial model diagram illustrating varicella vaccination compartments and transitions.

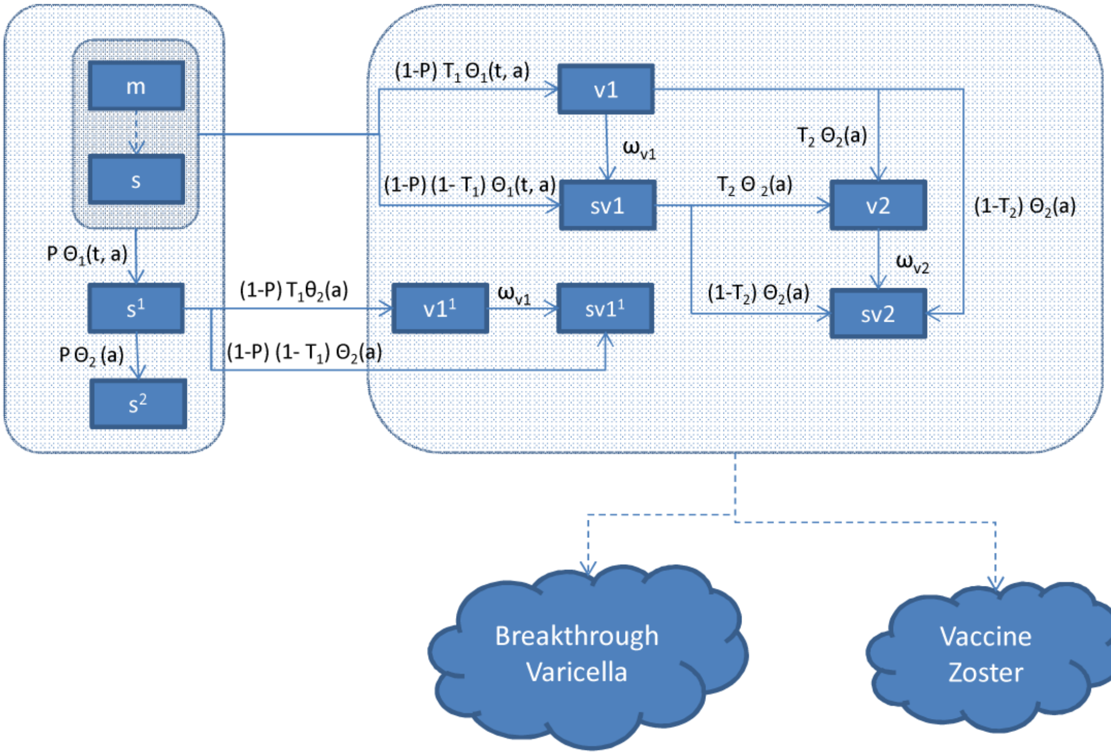

1.2.4. Breakthrough varicella

In the model, breakthrough varicella is defined as any case of varicella that arises in any successfully varicella-vaccinated person. The compartments and transitions are illustrated in Fig C.

Varicella-susceptible vaccinated individuals (sv1, sv1[1]) and (sv2) may acquire breakthrough varicella at age- and time-dependent rates $b_{v1}\lambda_a(t)$ and $b_{v2}\lambda_a(t)$ respectively, and become latently infected (evb), where $1 - b_{v1}$ and $1 - b_{v2}$are the vaccine degree of protection for first and second doses, respectively. Latently infected individuals become infectious (ivb) at a constant, age-independent rate ϵ_{vb}. Infectious individuals recover to fully HZ-protected (rvb) at a constant age-independent rate γ_{vb}. Full HZ immunity (rvb) wanes to HZ reactivation susceptible (wvb) at a constant, age-independent rate of δ_{vb}.

HZ reactivation susceptible individuals (wvb) may

- acquire wild-type HZ at the constant age-dependent rate $\chi\sigma(a)$, where χ is a factor reducing reactivation rate due to having been vaccinated with varicella or
- be boosted back to full HZ immunity (wvb) through exogenous boosting at an age- and time-dependent rate $\zeta_{vb}(a)\lambda_a(t)$ (exogenous boosting) and a constant, age-dependent rate $\xi_{vb}(a)$ (endogenous boosting)

High-immunity HZ and HZ-susceptible individuals (rvb, wvb) may be vaccinated with an HZ vaccine and

- acquire a degree of protection, $(1 - b_z)$, from HZ reactivation (vzb) in proportion T_z θ_z(a) to the aging rate or
- become susceptible to HZ reactivation (wvbz) in proportion $(1 - T_z)$ θ_z(a) to the aging rate.

Those who are partially protected from HZ reactivation (vzb) may acquire wild-type HZ (zwild) at an age-dependent rate b_z $\chi\sigma(a)$ or may become fully susceptible to HZ (wvbz) at a constant, age-independent rate ω_z. The fully susceptible individuals (wvbz) may acquire wild-type HZ (z) at an age-dependent rate $\chi\sigma(a)$. Wild-type HZ-infected individuals (zwild) recover (rz) at constant rate η_{vb} or die at a constant age-dependent rate $\text{cfr}_z(a)\eta_{vb}$ (Fig C).

Fig C. Breakthrough varicella: partial model diagram illustrating breakthrough varicella through wild zoster compartments and transitions.

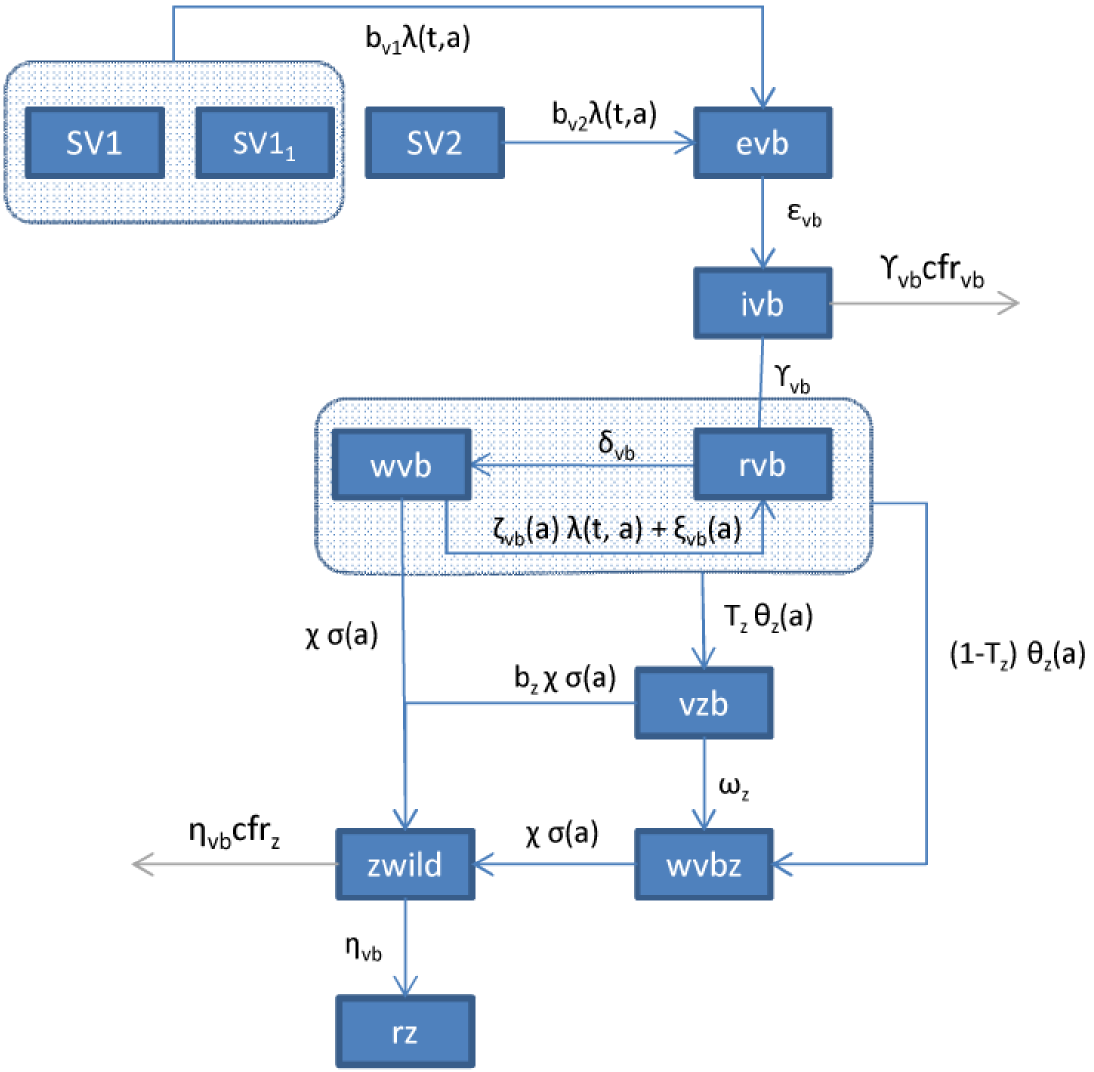

1.2.5. Vaccine herpes zoster

Fig D illustrates the model compartments and transitions from varicella vaccination through HZ susceptibility and vaccination. Individuals who are protected by one (v1, $v1^1$) or two (v2) successful varicella vaccine doses may become susceptible to HZ reactivation and permanent varicella immunity (wvv) at a constant, age-independent rate of π_1 or π_2, respectively. All varicella vaccine-protected individuals (v1, $v1^1$) and (v2) may be boosted to temporary full HZ immunity and permanent varicella immunity (rvv) at an age- and time-dependent rate of $k_{v1}\,\lambda(t,a)$ and $k_{v2}\,\lambda(t,a)$ respectively. Full HZ immunity (rvv) wanes to HZ reactivation susceptible (wvv) at a constant, age-independent rate of δ_{vv}.

HZ reactivation susceptible individuals (wvv) may

- acquire vaccine-type HZ at the constant age-dependent rate $\chi\sigma(a)$ or
- be boosted back to full HZ immunity (rvv) through exogenous boosting at an age- and time-dependent rate $\zeta_{vv}(a)\lambda_a(t)$ (exogenous boosting) and a constant, age-dependent rate $\xi_{vv}(a)$ (endogenous boosting)

All varicella-vaccinated, vaccinated-susceptible, high-immunity HZ, and HZ-susceptible individuals (v1, $v1^1$, v2, s1, $s1^1$, s2, rvv, wvv) may be vaccinated with an HZ vaccine and

- acquire a degree of protection, $(1-b_z)$, from HZ reactivation (vzv) in proportion $T_z\,\theta_z$(a) to the aging rate or
- become susceptible to vaccine-type HZ reactivation (wvvz) in proportion $(1-T_z)\,\theta_z$(a) to the aging rate

Those who are partially protected from HZ reactivation (vzv) may acquire vaccine-type HZ (zvacc) at an age-dependent rate $b_z\chi\,\sigma(a)$ or may become fully susceptible to HZ (wvvz) at a constant, age-independent rate ω_z. The fully susceptible individuals (wvvz) may acquire vaccine-type HZ (zvacc) at an age-dependent rate $\chi\,\sigma(a)$. Vaccine-type HZ-infected individuals (zvacc) recover (rz) at constant rate η_{vv} or die at a constant age-dependent rate of $\mathrm{cfr}_z(a)\eta_{vv}$.

Fig D. Vaccine zoster: partial model diagram illustrating from varicella vaccination through vaccine zoster transitions and compartments.

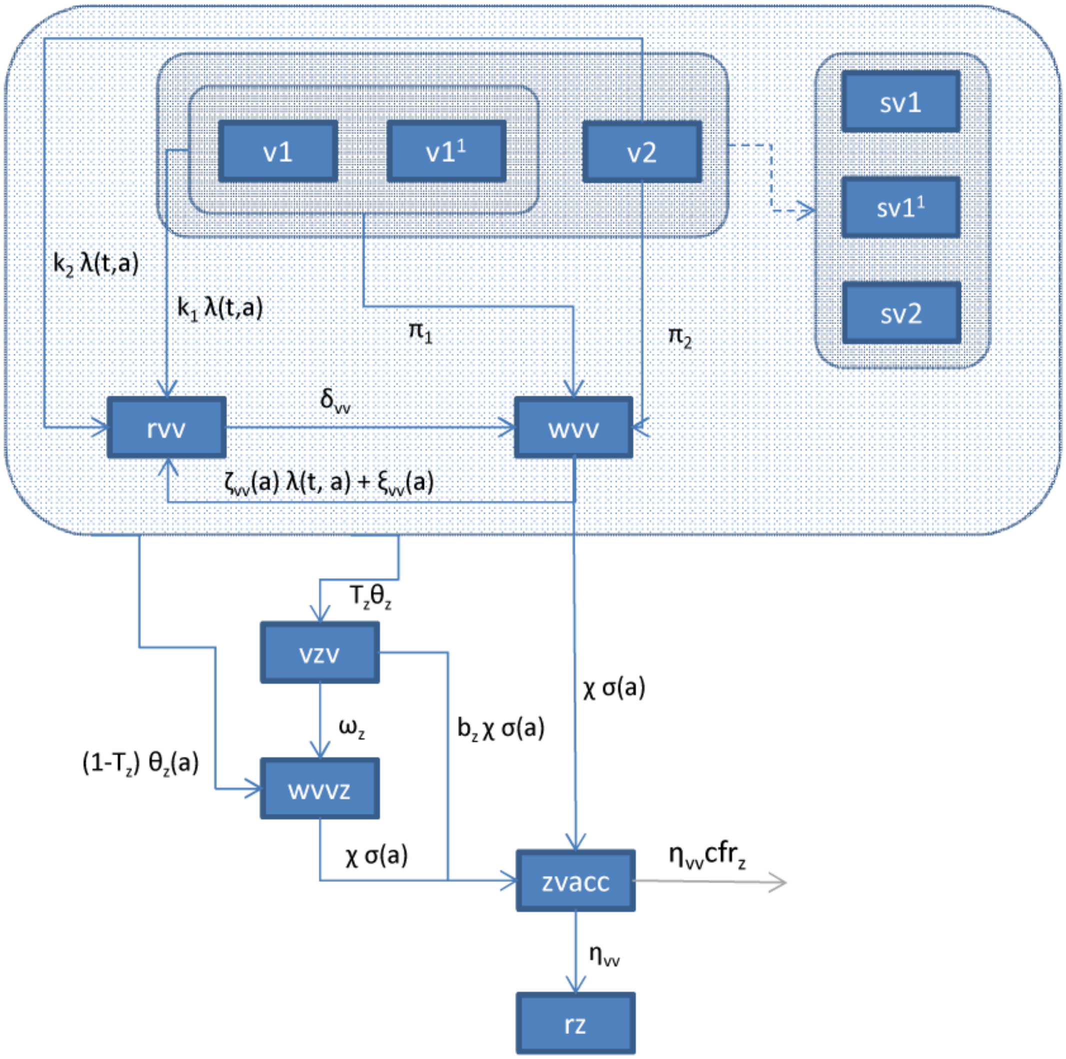

1.3. Model calibration

The model is calibrated to fit observed varicella data, using incidence or seroprevalence, and HZ incidence. Since the contribution of HZ to the overall force of infection is very small compared with the contribution from varicella disease, we can first calibrate the varicella force of infection from varicella data only and then calibrate for HZ incidence.

We calibrate the varicella model by finding a transmission matrix that fits the observed data. Calibration of the transmission matrix, $\beta_{k,j}$, requires making some assumptions about the structure of the matrix. Many different approaches have been taken by other authors [6,8,9,16,18,23-25] including assuming certain detailed Who acquires infection from Whom (WAIFW) approach whereby a mixing pattern is assumed and the variables defining the pattern are determined in calibration; using age-specific scaling of social contact matrix whereby the scale factors are determined in calibration; and proportionate mixing whereby the mixing matrix is assumed to be separable into a product of two mixing vectors, $\beta_{k,j} = c_k\ c_j$. We assume a fixed contact matrix as detailed in (Prem 2017) [26]

Since the model assumes a static population, it will produce a monotonically increasing seroprevalence profile, therefore, we approximate the Turkey seroprevalence data using a monotonically increasing function $F(x)$.

$$F(x) = \frac{a}{b + e^{c\,x-d}} + f \tag{6}$$

we fit this function using the left boundary of the age groups to arrive at the fitted form of the function specific for Turkey pre-vaccine seroprevalence (S2 Appendix, Table A**)**.

$$F(x) = \frac{1.035}{0.5825 + e^{0.2867\,x-0.4430}} + 0.96 \tag{7}$$

Since we are using a fixed contact matrix for Turkey [26], we need to adjust an age-specific susceptibility scpt_k to infection to calibrate the model. To do this we first find the equilibrium values of $\lambda_k(t)$ by fitting the "no vaccine" model seroprevalence to the observed seroprevalence for Turkey. Once we have the equilibrium forces of infection we can substitute the contact martix and the equilibrium compartment solutions into the right-hand side of equation (1) and the equilibrium force of infection into the left-hand side resulting in equation (8) where the $\bar{\lambda}_k, \bar{i}_j$, and $\bar{z}_j$ are equilibrium solutions to the no-vaccine model. These linear algebraic equations can be solved for the susceptibilities.

$$\bar{\lambda}_k = \sum_{j=1}^{ages} \mathrm{scpt}_k\, \omega_{k,j}\left[\bar{i}_j + \rho_z \bar{z}_j\right] \qquad \bar{\lambda}_k = \sum_{j=1}^{ages} c_k c_j\left[\bar{i}_j + \rho_z \bar{z}_j\right] \qquad (8)$$

The calibrated susceptibilities rates shown in S2 Appendix Table B were used to calculate the transmission matrix $\beta_{k,j} = \mathrm{scpt}_k\, \omega_{k,j}$.

. Calibration of the HZ model involves choosing which HZ-related transmission parameters to fit. There are at least three candidate parameters for fitting HZ:

1. Since we assume that exogenous boosting is proportional to the varicella force of infection we can adjust the effective contacts for exogenous boosting, $\zeta_n(a)$
2. **If** we allow for endogenous boosting to occur we can adjust the endogenous boosting rate, $\xi_n(a)$
3. **The** HZ reactivation rate, $\sigma_n(a)$.

For the base model we used the third approach along with the assumption that $\zeta_n(a)$=1. Thus we simply solve equation (9) for the $\sigma_k s$.where the $\bar{w}_k$ are the equilibrium HZ susceptible no-vaccine model population and $\hat{\mathrm{I}}_{HZ,k}$ are the known annual incidence of HZ from S2 Appendix, Table E. We then fit the commonly-used theoretical functional form for the reactivation rate,

$$\sigma(a) = \omega\, e^{-\varphi\, a} + \frac{\pi\, a^{\eta}}{100\ 000} \qquad (9)$$

A least-squares fit was done to find the parameter values, thus avoiding over-fitting and putting the model of HZ reactivation on firmer theoretical ground.

1.4. Model outcomes

1.4.1. Health outcomes

Annual incidence rate $I_{k,j}(t)$ for age group j is defined where "k" represents the final quantity, such as "natural varicella", "breakthrough varicella", "all varicella", "death", "vaccine dose",

etc., is generally as the transition rate from a particular set of health states appropriate for the "k" in question.

$$I_{k,j}(t) = \sum_{m}^{M} \epsilon_k^m X_{m,j}(t) \tag{10}$$

where ϵ_k^m is the transition rate from the mth contributing transition to the incidence k. Since the compartment population represents a proportion of the total population (all ages) we can get the the rates of combined age groups by summing over j.

For example, the total (all ages) incidence of natural varicella is given by

$$I_{natural}(t) = \sum_{j=1}^{ages} I_{natural,j}(t) = \sum_{j=1}^{ages}\sum_{m}^{M} \epsilon_k^m X_{m,j}(t) = \sum_{j=1}^{ages} \epsilon_n\left(e_j(t) + e_j^f(t)\right) \tag{11}$$

where $1/\epsilon_n$ is the duration of latent natural varicella infection, $e_j(t)$ is the unvaccinated exposed population in age group j, and $e_j^f(t)$ is the "failed vaccinated" exposed population for age group j. For breakthrough varicella there is only one contributing transition so

$$I_{breakthrough}(t) = \sum_{j=1}^{ages} I_{breakthrough,j}(t) = \epsilon_{vb} evb_j(t) \tag{12}$$

We easily get the total varicella (natural plus breakthrough) by adding equations (11) and (12). The number of cases is the cumulative incidence over a range of time so in general is

$$I_{k,j}(t_1,t_2) = \int_{t_1}^{t_2} dt \sum_{m}^{M} \epsilon_k^m E_{m,j}(t) \tag{13}$$

For example, the total cumulative incidence of all varicella, or number of cases, in one year starting at t=0 is

$$I_{all\ varicella}(0,1) = \int_{0}^{1} dt \sum_{j=1}^{ages} \epsilon_n\left(e_j(t) + e_j^f(t)\right) + \epsilon_{vb} evb_j(t) \tag{14}$$

The complete list of incidence outcomes is shown in the bulleted list below. For a given category, the sum of all of the stratifications gives the total incidence. As the list indicates, the HZ incidence category has two different stratifications. In addition the vaccine doses administered is essentially an incidence rate, that is, the instantaneous annual rate of doses administered and the actual number of doses administered in any given time period is the integral of the dose rate over that time period.

- Varicella incidence, comprising natural breakthrough, and congenital varicella
- HZ incidence, comprising wild-type and vaccine-type HZ
- HZ incidence, comprising uncomplicated HZ and PHN
- Deaths, comprising varicella-related and HZ-related deaths
- Varicella vaccine doses, comprising (if included) first, second, and catch-up doses

- HZ vaccine doses, comprising first doses (if included)

We also report the prevalence of susceptible individuals to natural or breakthrough varicella. In addition we report seroprevalence, an outcome intended for use in calibration that does not distinguish between vaccinated and non-vaccinated. Prevalence is a simple sum over compartment population size and results

$$P_{k,j}(t) = \sum_{m}^{M} X_{m,j}(t) \quad (15)$$

Where the X_m represent the appropriate set of compartments for the "k" in question. For example, the prevalence of natural varicella susceptible individuals at any given time in age group j is

$$P_{natural,j}(t) = s_j(t) + s_j^1(t) + s_j^2(t) \quad (16)$$

1.4.2. Economic outcomes

There is an extensive set of economic outputs from the model stratified at many levels. The top level outcomes are the costs, quality-adjusted life-years (QALYs), and health resource utilization.

Cost outcomes are also reported as instantaneous annual cost rates and are in general the result of multiplying incidence rates by unit costs and discounting. Cumulative costs are obtained by integrating the cost rates ($c_{k,l,m,n,j}$) over an appropriate time interval.

$$C_{k,l,m,n,j}(t) = c_{k,l,m,n,j} e^{-r_c t} I_{k,j}(t) \quad (17)$$

Where the indices, k, l, m, and n, represent successive levels of stratification and r_c is the discount rate to be applied to costs. The levels of stratification for cost outcomes are detailed in Table D.

The total cost for any level is obtained by summing over all the substrata. For example, the total treatment (TC) cost for natural varicella (NV) for all ages is given by

$$C_{TC,NV}(t) = \sum_{j=1}^{ages} e^{-r_c t} I_{NV,j}(t) \sum_{m}^{\{dc,ic\}} \sum_{n}^{\{op,ip\}} c_{TC,NV,m,n,j} \quad (18)$$

where "dc", "ic","op", and "ip" represent direct, indirect, outpatient, and inpatient costs respectively. Cumulative costs are obtained by integrating equation (17) over the appropriate time interval as below

$$C_{k,l,m,n,j}(t_1, t_2) = \int_{t_1}^{t_2} dt\, c_{k,l,m,n,j} e^{-r_c t} I_{k,j}(t) \quad (19)$$

Table D. Levels of cost stratification for outcomes.

Category (k)	Dose stratification (l)	Cost type (m)	Cost type (n)
Vaccination Costs	1st dose	Direct	N/A
		Indirect	
	2nd dose	Direct	N/A
		Indirect	
	catch-up dose	Direct	N/A
		Indirect	
	HZ dose	Direct	N/A
		Indirect	
Treatment Costs	Natural Varicella	Direct	Outpatient
			Inpatient
		Indirect	Outpatient
			Inpatient
	Breakthrough Varicella	Direct	Outpatient
			Inpatient
		Indirect	Outpatient
			Inpatient
	Wild-type HZ	Direct	Uncomplicated
			PHN
		Indirect	Uncomplicated
			PHN
	Vaccine-type HZ	Direct	Uncomplicated
			PHN
		Indirect	Uncomplicated
			PHN

Quality-adjusted life-years (QALYs) are calculated as the sum of life-years lost due to disease and QALY decrements relative to perfect health. The model outcomes stratifies QALY decrements into four levels: (1) natural varicella, (2) breakthrough varicella, (3) vaccine-type HZ, and (4) wild-type HZ. QALYs are also discounted at a rate r_q. QALYs are calculated from the model as follows

$$QALY\ decrement_{NV,j}(t_1,t_2) = q_{h,j}\int_{t_1}^{t_2} dt\,(1-q_{n,j})\,e^{-r_q t} i_j(t) + LifeYearsLost_j \int_{t_1}^{t_2} dt \mu_{NV} i_j(t) \tag{20}$$

$$QALY\ decrement_{BV,j}(t_1,t_2) = q_{h,j}\int_{t_1}^{t_2} dt\,(1-q_{bv,j})\,e^{-r_q t} ivb_j(t) + LifeYearsLost_j \int_{t_1}^{t_2} dt \mu_{BV} i_j(t) \tag{21}$$

$$\begin{aligned} &QALY\ decrement_{WHZ,j}(t_1,t_2) \\ &= q_{h,j}\int_{t_1}^{t_2} dt\, e^{-r_q t}\Big\{\Big((1-q_{z,j})(1-p_{phn}) + d_{phn}\,\eta_n(1-q_{phn,j})p_{phn}\Big)\, z_j(t) \\ &+ \Big((1-q_{z,j})(1-p_{phn}) \\ &+ d_{phn}\,\eta_{nvb}(1-q_{phn,j})p_{phn}\Big)\, zwild_j(t)\Big\} + LifeYearsLost_j \int_{t_1}^{t_2} dt \mu_{HZ}\big(z_j(t) + zwild_j(t)\big) \end{aligned} \tag{22}$$

$$\begin{aligned} &QALY\ decrement_{VHZ,j}(t_1,t_2) \\ &= q_{h,j}\int_{t_1}^{t_2} dt\, e^{-r_q t}\Big\{\Big((1-q_{zv,j})(1-p_{phn}) + d_{phn}\,\eta_n(1-q_{phn,j})p_{phn}\Big)\, zvacc_j(t) \\ &+ \Big((1-q_{z,j})(1-p_{phn}) \\ &+ d_{phn}\,\eta_{nvb}(1-q_{phn,j})p_{phn}\Big)\, zwild_j(t)\Big\} + LifeYearsLost_j \int_{t_1}^{t_2} dt \mu_{HZ}\, zvacc_j(t) \end{aligned} \tag{23}$$

$$LifeYearsLost_j = \int_0^{\infty} dt e^{r_q t} \sum_i q_{h,i} p_i(t)\,, \qquad \begin{cases} p_{i<j}(t) = 0 \\ p_{i=j}(0) = 1 \\ p_{i>j}(0) = 0 \end{cases} \tag{24}$$

Where the "q"s are the age-dependent health utilities for various health states and are detailed in Table E. The QALY calculations for HZ are complicated by the fact that we do not track PHN/non-PHN HZ in separate compartments. Thus, the contribution of each of the compartments contributing to HZ disutility is split into the proportion of individuals with PHN and that without PHN. The PHN contribution has to be further scaled to take into account that the duration of PHN is substantially longer than the duration of non-PHN HZ by multiplying by $d_{phn}\ \eta_{nvb}$. This is the ratio of the durations of PHN and non-PHN HZ disease.

Table E. Health state utility values used in the model [27].

Health state	Utility by age (years)					
Healthy (q_h)	Ages	<40	40 - 60	60 - 70	70 - 80	≥80
	Utility	1	0.901	0.871	0.833	0.729
Natural varicella (q_n)	Ages	<15	≥15			
	Utility	0.81	0.73			
Breakthrough varicella (q_{vv})	Ages	<15	≥15			
	Utility	0.905	0.865			
Uncomplicated HZ (q_{zv})	Ages	All				
	Utility	0.73				
PHN Zoster (q_{phn})	Ages	All				
	Utility	0.671				

1.5. Probabilistic sensitivity analyses

In addition to one-way sensitivity analyses, we performed probabilistic sensitivity analyses.

Gamma distribution is appropriate for non-negative, skewed data sets. Hence, because costs are constrained to be non-negative and are highly skewed, the gamma distribution was chosen for representing uncertainty in cost parameters [28]. In addition, the parameter representing median duration of protection is constrained between 0 and infinity, suggesting gamma as a reasonable distribution for representing uncertainty regarding duration of protection. The parameters of the gamma distribution were obtained using the method of moments. Given the mean (*m*), low (*l*), and high (*h*) values of the parameter as shown in Table C, we estimate the standard error (se) as

$$se = \frac{h - l}{2(1.96)}$$

The parameters of the Gamma distribution are given by

$$\alpha = \frac{m^2}{se^2},$$
$$\beta = \frac{se^2}{m}$$

Beta distributions are appropriate for values restricted between 0 and 1. Therefore, because utilities or health-related quality of life (QoL) weights in this analysis are assumed to be between 0 and 1, we used a beta distribution to represent uncertainty in QoL parameters. Base case values were used as mean of beta distribution and standard error was estimated using the equation below. In addition, beta distribution is a reasonable choice for representing uncertainty in vaccine uptake parameters because vaccine uptake in the model was represented by a

proportion of persons moving to older age groups that can take a value between 0 and 1. The parameters for the beta distribution were obtained as

$$\alpha = \frac{m(m - m^2 - se^2)}{se^2},$$

$$\beta = \frac{(1 - m)(m - m^2 - se^2)}{se^2}$$

The properties of the vaccine include degree of protection against infection, and degree of protection against disease given a breakthrough infection. The degree of protection is 1 minus residual susceptibility (i.e., relative risk of infection among vaccine and placebo recipients). Because the relative risk ranges from zero to infinity, the appropriate distribution for residual susceptibility parameters is a log-normal distribution [28]. Because the mean relative risk is zero, we used a derived distribution to represent the uncertainty in degree of protection against infection.

Table F. Probabilistic sensitivity analysis parameters.

Parameter	Base	Low	High	Distribution
PrimaryEfficacy	100	80	100	LogNormal(-4.6052,1.5285)
BoosterEfficacy	100	80	100	LogNormal(-4.6052,1.5285)
DurationOfVaccineImmunityDose1	25	20	30	Gamma(96.04,0.26031)
HZSusceptibleTransitionRate1	25	0.033333	0.05	Gamma(96.04,0.26031)
DurationOfVaccineImmunityDose2	77	61.6	92.4	Gamma(96.04,0.80175)
HZSusceptibleTransitionRate2	77	0.010823	0.016234	Gamma(96.04,0.80175)
VaccinatedSusceptibility1	100	80	100	LogNormal(-4.6052,1.5285)
VaccinatedSusceptibility2	100	80	100	LogNormal(-4.6052,1.5285)
BoostingProbability1	100	80	100	Beta(30.217,3.3574)
BoostingProbability2	100	80	100	Beta(30.217,3.3574)
VaccineTake	96	76.8	100	Beta(24.996,3.28)
QALYWeightsBreakthroughVaricella$Raw	0.905	0.724	0.967301	LogNormal(-2.3539,0.54415)
QALYWeightsNaturalVaricella$Raw	0.81	0.648	0.897443	LogNormal(-1.6607,0.3146)
QALYWeightsBreakthroughZoster$Raw	0.73	0.584	0.82476	LogNormal(-1.3093,0.22055)
QALYWeightsPHN$Raw	0.671	0.5368	0.766319	LogNormal(-1.1117,0.17454)
QALYWeightsZoster$Raw	0.73	0.584	0.82476	LogNormal(-1.3093,0.22055)
ReactivationRateVaccineFactor	0.166667	0.083333	0.25	LogNormal(-1.9356,0.28026)
VaricellaDoseCosts	50.55	40.44	60.66	Gamma(96.04,0.52634)
VaricellaWorkDaysLostOP*	2.5	2	3	Gamma(15.366,0.16269)
VaricellaWorkDaysLostIP*	7.768333	6.214667	9.322	Gamma(96.04,0.080886)
CostPerWorkDayMissed	55.74	44.592	66.888	Gamma(96.04,0.58038)
VaricellaVaccineWorkDaysLost*	0.26	0.208	0.312	Gamma(96.04,0.0027072)
VaricellaCarePercentOP*	0.9	0.72	1	Beta(19.435,3.1638)
VaricellaCareUtilizationOP*	2	1.6	2.4	Gamma(96.04,0.020825)
VaricellaCostForCareOP*	55	44	66	Gamma(96.04,0.57268)
VaricellaCarePercentIP*	0.072186	0.057749	0.086623	Beta(89.035,1144.4)
VaricellaCareUtilizationIP*	6.811429	5.449143	8.173714	Gamma(96.04,0.070923)
VaricellaCostForCareIP*	92.91	74.328	111.492	Gamma(96.04,0.96741)
VaricellaMedicationCountOP*	3	2.4	3.6	Gamma(96.04,0.031237)
VaricellaMedicationPercentOP*	0.97	0.8	1	Beta(9.8737,0.30537)
VaricellaAverageDrugCostOutpatient*	11.06	8.848	13.272	Gamma(96.04,0.11516)
ZosterCostForCareUN*	873.2538	698.603	1047.905	Gamma(96.04,9.0926)
ZosterCostForCarePHN*	1883.185	1506.548	2259.823	Gamma(96.04,19.608)
ZosterCarePercentUN*	100	0.784	0.9999	Beta(5.3521,0.10923)
ZosterCarePercentPHN*	100	0.784	0.9999	Beta(5.3521,0.10923)
PrimaryCoverage	95	76	95	Beta(33.579,3.731)
BoosterCoverage	90	72	95	Beta(22.629,2.5143)

* age-dependent variables; reference age group case for sensitivity analysis shown.

1.6. Differential equations for the model

$$
\begin{aligned}
m_j{}'(t) &= \left(\Lambda - \Lambda_c(t) - \Lambda_s(t)\right)\boldsymbol{\delta}_{j,1} + d_{j-1}m_{j-1}(t) - \left(\theta_{c_j} + \theta_{1_j} + \theta_{s_j}\right) d_{j-1}m_{j-1}(t) - \left(d_j + \omega_{\mathrm{m}} + \mu_j\right)m_j(t) \\
\mathrm{v1}_j{}'(t) &= d_{j-1}\mathrm{v1}_{j-1}(t) + P\,T_1\left(\theta_{c_j} + \theta_{1_j} + \theta_{s_j}\right) d_{j-1}m_{j-1}(t) + P\,T_1\left(\theta_{c_j} + \theta_{1_j} + \theta_{s_j}\right) d_{j-1}\,s_{j-1}(t) \\
&\quad - \left(\theta_{2_j} + \theta_{z_j}\right) d_{j-1}\mathrm{v1}_{j-1}(t) - \left(d_j + k_1\lambda_j(t) + \omega_{\mathrm{v}_1} + \mu_j + \pi_1\right)\mathrm{v1}_j(t) \\
\mathrm{v1}_j^{1\prime}(t) &= d_{j-1}\,v1_{j-1}^1(t) + PT_1 d_{j-1}\theta_{2_j}s_{j-1}^1 - d_{j-1}\theta_{z_j}\,v1_{j-1}^1(t) - \left(d_j + k_1\lambda_j(t) + \omega_{\mathrm{v}_1} + \mu_j + \pi_1\right) v1_j^1(t) \\
\mathrm{v2}_j{}'(t) &= d_{j-1}\mathrm{v2}_{j-1}(t) + T_2\theta_{2_j}d_{j-1}\mathrm{sv1}_{j-1}(t) + T_2\theta_{2_j}d_{j-1}\mathrm{v1}_{j-1}(t) - \theta_{z_j}d_{j-1}\mathrm{v2}_{j-1}(t) \\
&\quad -\left(d_j + k_2\lambda_j(t) + \omega_{\mathrm{v}_2} + \mu_j + \pi_2\right)\mathrm{v2}_j(t) \\
\mathrm{vzv}_j{}'(t) &= d_{j-1}\mathrm{vzv}_{j-1}(t) + T_z\theta_{z_j}d_{j-1}\,sv1_{j-1}^1(t) + T_z\theta_{z_j}\,d_{j-1}v1_{j-1}^1(t) + d_{j-1}T_z\mathrm{rvv}_{j-1}(t)\theta_{z_j} \\
&\quad + T_z\theta_{z_j}d_{j-1}\left(\mathrm{sv1}_{j-1}(t) + \mathrm{sv2}_{j-1}(t) + \mathrm{v1}_{j-1}(t) + \mathrm{v2}_{j-1}(t) + \mathrm{wvv}_{j-1}(t)\right) \\
&\quad - \left(d_j + \chi b_z\sigma_j + \mu_j + \omega_z\right)\mathrm{vzv}_j(t) \\
\mathrm{vzw}_j{}'(t) &= d_{j-1}\mathrm{vzw}_{j-1}(t) + T_z\theta_{z_j}d_{j-1}\left(r_{j-1}(t) + \mathrm{rc}_{j-1}(t) + w_{j-1}(t)\right) - \left(d_j + b_z\sigma_j + \mu_j + \omega_z\right)\mathrm{vzw}_j(t) \\
\mathrm{vzb}_j{}'(t) &= d_{j-1}\mathrm{vzb}_{j-1}(t) + T_z\theta_{z_j}d_{j-1}\left(\mathrm{rvb}_{j-1}(t) + \mathrm{wvb}_{j-1}(t)\right) - \left(d_j + \chi b_z\sigma_j + \mu_j + \omega_z\right)\mathrm{vzb}_j(t) \\
s_j{}'(t) &= \Lambda_s(t)\boldsymbol{\delta}_{j,1} + d_{j-1}s_{j-1}(t) + \omega_{\mathrm{m}}m_j(t) - \left(\theta_{c_j} + \theta_{s_j} + \theta_{1_j}\right) d_{j-1}s_{j-1}(t) - \left(d_j + \lambda_j(t) + \mu_j\right)s_j(t) \\
\mathrm{s}_j^{1\prime}(t) &= d_{j-1}\,\mathrm{s}_{j-1}^1(t) + (1-P)\left(\theta_{c_j} + \theta_{s_j} + \theta_{1_j}\right) d_{j-1}m_{j-1}(t) + (1-P)\left(\theta_{c_j} + \theta_{s_j} + \theta_{1_j}\right) d_{j-1}s_{j-1}(t) \\
&\quad -\theta_{2_j}\,d_{j-1}\mathrm{s}_{j-1}^1(t) - \left(d_j + \lambda_j(t) + \mu_j\right)\mathrm{s}_j^1(t) \\
\mathrm{s}_j^{2\prime}(t) &= d_{j-1}s^2{}_{j-1}(t) + (1-P)\theta_{2_j}d_{j-1}s^1{}_{j-1}(t) - \left(d_j + \lambda_j(t) + \mu_j\right)s^2{}_j(t) \\
\mathrm{sv1}_j{}'(t) &= d_{j-1}\mathrm{sv1}_{j-1}(t) + \omega_{\mathrm{v}_1}\mathrm{v1}_j(t) + P(1-T_1)\left(\theta_{s_j} + \theta_{1_j} + \theta_{c_j}\right) d_{j-1}m_{j-1}(t) \\
&\quad + P(1-T_1)\left(\theta_{s_j} + \theta_{1_j} + \theta_{c_j}\right) d_{j-1}s_{j-1}(t) - \left(\theta_{2_j} + \theta_{z_j}\right) d_{j-1}\mathrm{sv1}_{j-1}(t) \\
&\quad - \left(d_j + b_{\mathrm{v}_1}\lambda_j(t) + \mu_j\right)\mathrm{sv1}_j(t) \\
\mathrm{sv1}_j^{1\prime}(t) &= d_{j-1}\,\mathrm{sv1}_{j-1}^1(t) + \omega_{\mathrm{v}_1}\,\mathrm{sv1}_j^1(t) + P(1-T_1)\,\theta_{2_j}d_{j-1}\mathrm{s}_{j-1}^1(t) - \theta_{z_j}\,d_{j-1}\mathrm{sv1}_{j-1}^1(t) \\
&\quad -\left(d_j + b_{\mathrm{v}_1}\lambda_j(t) + \mu_j\right)\mathrm{sv1}_j^1(t) \\
\mathrm{sv2}_j{}'(t) &= d_{j-1}\mathrm{sv2}_{j-1}(t) + +\omega_{\mathrm{v}_2}\mathrm{v2}_j(t) + (1-T_2)d_{j-1}\theta_{2_j}(\mathrm{sv1}_{j-1}(t) + \mathrm{v1}_{j-1}(t)) - \theta_{z_j}d_{j-1}\mathrm{sv2}_{j-1}(t) \\
&\quad -\left(b_{\mathrm{v}_2}\lambda_j(t) + -d_j + \mu_j\right)\mathrm{sv2}_j(t)
\end{aligned}
$$

$$
\begin{aligned}
e_j{}'(t) &= d_{j-1}e_{j-1}(t) + s_j(t)\lambda_j(t) - \left(dj + \epsilon_\mathrm{n} + \mu_j\right)e_j(t)\\
e^f{}_j{}'(t) &= d_{j-1}e^f{}_{j-1}(t) + \lambda_j(t)\left(s^1{}_j(t) + s^2{}_j(t)\right) - \left(d_j + \epsilon_\mathrm{n} + \mu_j\right)e^f{}_j(t)\\
\mathrm{evb}_j{}'(t) &= d_{j-1}\mathrm{evb}_{j-1}(t) + \lambda_j(t)\left(b_{\mathrm{v}_1}\mathrm{sv1}_j(t) + b_{\mathrm{v}_2}\mathrm{sv2}_j(t) + b_{\mathrm{v}_1}\,\mathrm{sv1}^1{}_j(t)\right) - \left(d_j + \epsilon_\mathrm{vb} + \mu_j\right)\mathrm{evb}_j(t)\\
i_j{}'(t) &= d_{j-1}i_{j-1}(t) + \epsilon_\mathrm{n}e_j(t) + \epsilon_\mathrm{n}e^f{}_j(t) - \left(d_j + \mathrm{cfr}_{\mathrm{n}\,j}\gamma_\mathrm{n} + \gamma_\mathrm{n} + \mu_j\right)\, i_j(t)\\
\mathrm{ivb}_j{}'(t) &= d_{j-1}\mathrm{ivb}_{j-1}(t) + \epsilon_\mathrm{vb}\mathrm{evb}_j(t) - \left(d_j + \mathrm{cfr}_{\mathrm{vb}\,j}\gamma_\mathrm{vb} + \gamma_\mathrm{vb} + \mu_j\right)\mathrm{ivb}_j(t)\\
r_j{}'(t) &= d_{j-1}r_{j-1}(t) + \gamma_\mathrm{n}i_j(t) + \left(\zeta_{\mathrm{n}\,j}\lambda_j(t) + \xi_{\mathrm{n}\,j}\right)w_j(t) - d_{j-1}r_{j-1}(t)\theta_{\mathrm{z}\,j} - \left(d_j + \delta_\mathrm{n} + \mu_j\right)r_j(t)\\
\mathrm{rc}_j{}'(t) &= \Lambda_c(t)\boldsymbol{\delta}_{j,1} + d_{j-1}\mathrm{rc}_{j-1}(t) - d_{j-1}\mathrm{rc}_{j-1}(t)\theta_{\mathrm{z}\,j} - \left(d_j + \delta_\mathrm{n} + \mu_j\right)\mathrm{rc}_j(t)\\
\mathrm{rvb}_j{}'(t) &= d_{j-1}\mathrm{rvb}_{j-1}(t) + \gamma_\mathrm{vb}\mathrm{ivb}_j(t) + \left(\zeta_{\mathrm{vb}\,j}\lambda_j(t) + \xi_{\mathrm{vb}\,j}\right)\mathrm{wvb}_j(t) - d_{j-1}\mathrm{rvb}_{j-1}(t)\theta_{\mathrm{z}\,j}\\
&\quad - \left(d_j + \delta_\mathrm{vb} + \mu_j\right)\mathrm{rvb}_j(t)\\
\mathrm{rvv}_j{}'(t) &= d_{j-1}\mathrm{rvv}_{j-1}(t) + \lambda_j(t)\left(k_1\mathrm{v1}_j(t) + k_1\,\mathrm{v1}_j^1(t) + k_2\mathrm{v2}_j(t)\right) + \left(\zeta_{\mathrm{vv}\,j}\lambda_j(t) + \xi_{\mathrm{vv}\,j}\right)\mathrm{wvv}_j(t)\\
&\quad \theta_{\mathrm{z}\,j}d_{j-1}\mathrm{rvv}_{j-1}(t) - \left(d_j + \mu_j + \delta_\mathrm{vv}\right)\mathrm{rvv}_j(t)\\
w_j{}'(t) &= d_{j-1}w_{j-1}(t) + \delta_\mathrm{n}\left(r_j(t) + \mathrm{rc}_j(t)\right) - \theta_{\mathrm{z}\,j}d_{j-1}w_{j-1}(t) - \left(\zeta_{\mathrm{n}\,j}\lambda_j(t) + \xi_{\mathrm{n}\,j} + d_j + \sigma_j + \mu_j\right)w_j(t)\\
\mathrm{wvb}_j{}'(t) &= d_{j-1}\mathrm{wvb}_{j-1}(t) + \delta_\mathrm{vb}\mathrm{rvb}_j(t) - \theta_{\mathrm{z}\,j}d_{j-1}\mathrm{wvb}_{j-1}(t)\\
&\quad - \left(\zeta_{\mathrm{vb}\,j}\lambda_j(t) + \xi_{\mathrm{vb}\,j} + +\sigma_j + \mu_j + \chi\sigma_j + \mu_j\right)\mathrm{wvb}_j(t)\\
\mathrm{wvv}_j{}'(t) &= d_{j-1}\mathrm{wvv}_{j-1}(t) + \delta_\mathrm{vv}\mathrm{rvv}_j(t) + \pi_1\,\mathrm{v1}_j^1(t) + \pi_1\mathrm{v1}_j(t) + \pi_2\mathrm{v2}_j(t) - \theta_{\mathrm{z}\,j}d_{j-1}\mathrm{wvv}_{j-1}(t)\\
&\quad - \left(\zeta_{\mathrm{vv}\,j}\lambda_j(t) + \xi_{\mathrm{vv}\,j} + +\sigma_j + \mu_j + \chi\sigma_j + \mu_j\right)\mathrm{wvv}_j(t)\\
\mathrm{wz}_j{}'(t) &= d_{j-1}\mathrm{wz}_{j-1}(t) + \omega_\mathrm{z}\mathrm{vzw}_j(t) + (1 - T_\mathrm{z})\theta_{\mathrm{z}\,j}d_{j-1}\left(r_{j-1}(t) + \mathrm{rc}_{j-1}(t) + w_{j-1}(t)\right)\\
&\quad -\left(d_j + \omega_\mathrm{z} + \sigma_j + \mu_j\right)\mathrm{wz}_j(t)\\
\mathrm{wvbz}_j{}'(t) &= d_{j-1}\mathrm{wvbz}_{j-1}(t) + \omega_\mathrm{z}\mathrm{vzb}_j(t) + (1 - T_\mathrm{z})\theta_{\mathrm{z}\,j}d_{j-1}\left(\mathrm{rvb}_{j-1}(t) + \mathrm{wvb}_{j-1}(t)\right)\\
&\quad - \left(d_j + \chi\sigma_j + \mu_j\right)\mathrm{wvbz}_j(t)\\
\mathrm{wvvz}_j{}'(t) &= d_{j-1}\mathrm{wvvz}_{j-1}(t) + \theta_{\mathrm{z}\,j}(1 - T_\mathrm{z})d_{j-1}\left[sv1^1{}_{j-1}(t) + v1^1{}_{j-1}(t) + \mathrm{rvv}_{j-1}(t) + \mathrm{sv1}_{j-1}(t) + \mathrm{sv2}_{j-1}(t)\right.\\
&\quad \left. +\ \mathrm{v1}_{j-1}(t) + \mathrm{v2}_{j-1}(t) + \mathrm{wvv}_{j-1}(t)\right] - \left(d_j + \omega_\mathrm{z} + \mu_j + \chi\sigma_j\right)\mathrm{wvvz}_j(t)\\
z_j{}'(t) &= d_{j-1}z_{j-1}(t) + \sigma_j\left(b_\mathrm{z}\mathrm{vzw}_j(t) + w_j(t) + \mathrm{wz}_j(t)\right) - \left(d_j + \mathrm{cfr}_{z\,j}\eta_\mathrm{n} + \eta_\mathrm{n} + \mu_j\right)z_j(t)\\
\mathrm{zvacc}_j{}'(t) &= d_{j-1}\mathrm{zvacc}_{j-1}(t) + \chi\sigma_j\left(b_\mathrm{z}\mathrm{vzv}_j(t) + \mathrm{wvv}_j(t) + \mathrm{wvvz}_j(t)\right) - \left(d_j + \eta_\mathrm{vv} + \mu_j\right)\mathrm{zvacc}_j(t)\\
\mathrm{zwild}_j{}'(t) &= d_{j-1}\mathrm{zwild}_{j-1}(t) + \chi\sigma_j\left(b_\mathrm{z}\mathrm{vzb}_j(t) + \mathrm{wvb}_j(t) + \mathrm{wvbz}_j(t)\right) - \left(d_j + \eta_\mathrm{vb} + \mu_j\right)\mathrm{zwild}_j(t)\\
\mathrm{rz}_j{}'(t) &= d_{j-1}\mathrm{rz}_{j-1}(t) + \eta_\mathrm{n}z_j(t) + \eta_\mathrm{vb}\mathrm{zwild}_j(t) + \eta_\mathrm{vv}\mathrm{zvacc}_j(t) - \left(d_j + \mu_j\right)\mathrm{rz}_j(t)
\end{aligned}
$$

Where $\boldsymbol{\delta}_{j,1} = \begin{cases} 1\,; j = 1 \\ 0\,; otherwise \end{cases}$, $d_0 = 0$, $d_{ages} = 0$ and $1 < j \le ages$.

References

1. Hethcote HW. An age-structured model for pertussis transmission. Math Biosci. 1997;145:89-136.
2. Gershon AA, Raker R, Steinberg S, Topf-Olstein B, Drusin LM. Antibody to Varicella-Zoster virus in parturient women and their offspring during the first year of life. Pediatrics. 1976;58:692-696.
3. Gordon JE, Meader FM. The period of infectivity and serum prevention of chickenpox. JAMA. 1929;93:2013-2015. doi: 10.1001/jama.1929.02710260001001.
4. Izurieta HS, Strebel PM, Blake PA. Postlicensure effectiveness of varicella vaccine during an outbreak in a child care center. JAMA. 1997;278:1495-1499.
5. Garnett GP, Grenfell BT. The epidemiology of varicella-zoster virus infections: a mathematical model. Epidemiol Infect. 1992;108:495-511.
6. Poletti P, Melegaro A, Ajelli M, Del Fava E, Guzzetta G, Faustini L, et al. Perspectives on the impact of varicella immunization on herpes zoster. A model-based evaluation from three European countries. PLoS One. 2013;8:e60732. doi: 10.1371/journal.pone.0060732.
7. Coudeville L, Brunot A, Szucs TD, Dervaux B. The economic value of childhood varicella vaccination in France and Germany. Value Health. 2005;8:209-222. doi: 10.1111/j.1524-4733.2005.04005.x.
8. Schuette MC, Hethcote HW. Modeling the effects of varicella vaccination programs on the incidence of chickenpox and shingles. Bull Math Biol. 1999;61:1031-1064.
9. Brisson M, Edmunds WJ, Gay NJ, Law B, De Serres G. Modelling the impact of immunization on the epidemiology of varicella zoster virus. Epidemiol Infect. 2000;125:651-669.
10. United Nations Department of Economic and Social Affairs Population Division. Births by age of mother 2017 [cited May 9 2019]. Available from: https://esa.un.org/unpd/wpp/DVD/Files/1_Indicators (Standard)/EXCEL_FILES/2_Fertility/WPP2017_FERT_F06_BIRTHS_BY_AGE_OF_MOTHER.xlsx.
11. Edmunds WJ, Brisson M, Rose JD. The epidemiology of herpes zoster and potential cost-effectiveness of vaccination in England and Wales. Vaccine. 2001;19:3076-3090.
12. Dinleyici EC, Kurugol Z, Kara A, Tezer H, Tas MA, Guler E, et al. Children with breakthrough varicella infection requiring hospitalization in Turkey (VARICOMP Study 2008-2013). Vaccine. 2015;33:3983-3987. doi: 10.1016/j.vaccine.2015.06.029.

13. Valentim J, Sartori AM, de Soarez PC, Amaku M, Azevedo RS, Novaes HM. Cost-effectiveness analysis of universal childhood vaccination against varicella in Brazil. Vaccine. 2008;26:6281-6291. doi: 10.1016/j.vaccine.2008.07.021.
14. Harger JH, Ernest JM, Thurnau GR, Moawad A, Thom E, Landon MB, et al. Frequency of congenital varicella syndrome in a prospective cohort of 347 pregnant women. Obstet Gynecol. 2002;100:260-265.
15. Brisson M, Edmunds WJ. Varicella vaccination in England and Wales: cost-utility analysis. Arch Dis Child. 2003;88:862-869.
16. van Hoek AJ, Melegaro A, Zagheni E, Edmunds WJ, Gay N. Modelling the impact of a combined varicella and zoster vaccination programme on the epidemiology of varicella zoster virus in England. Vaccine. 2011;29:2411-2420. doi: 10.1016/j.vaccine.2011.01.037.
17. Tseng HF, Harpaz R, Luo Y, Hales CM, Sy LS, Tartof SY, et al. Declining effectiveness of herpes zoster vaccine in adults aged >/=60 years. J Infect Dis. 2016;213:1872-1875. doi: 10.1093/infdis/jiw047.
18. Brisson M, Melkonyan G, Drolet M, De Serres G, Thibeault R, De Wals P. Modeling the impact of one- and two-dose varicella vaccination on the epidemiology of varicella and zoster. Vaccine. 2010;28:3385-3397. doi: 10.1016/j.vaccine.2010.02.079.
19. McLean AR, Blower SM. Imperfect vaccines and herd immunity to HIV. Proc Biol Sci. 1993;253:9-13. doi: 10.1098/rspb.1993.0075.
20. Lamont RF, Sobel JD, Carrington D, Mazaki-Tovi S, Kusanovic JP, Vaisbuch E, et al. Varicella-zoster virus (chickenpox) infection in pregnancy. BJOG. 2011;118:1155-1162. doi: 10.1111/j.1471-0528.2011.02983.x.
21. Pupco A, Bozzo P, Koren G. Herpes zoster during pregnancy. Can Fam Physician. 2011;57:1133.
22. Hethcote HW. The mathematics of infectious diseases. SIAM Rev. 2000;42:599-653. doi: https://doi.org/10.1137/S0036144500371907.
23. Betta M, Laurino M, Pugliese A, Guzzetta G, Landi A, Manfredi P. Perspectives on optimal control of varicella and herpes zoster by mass routine varicella vaccination. Proc Biol Sci. 2016;283:20160054. doi: 10.1098/rspb.2016.0054.
24. Horn J, Karch A, Damm O, Kretzschmar ME, Siedler A, Ultsch B, et al. Current and future effects of varicella and herpes zoster vaccination in Germany - Insights from a

mathematical model in a country with universal varicella vaccination. Hum Vaccin Immunother. 2016;12:1766-1776. doi: 10.1080/21645515.2015.1135279.

25. Marziano V, Poletti P, Guzzetta G, Ajelli M, Manfredi P, Merler S. The impact of demographic changes on the epidemiology of herpes zoster: Spain as a case study. Proc Biol Sci. 2015;282:20142509. doi: 10.1098/rspb.2014.2509.

26. Prem K, Cook AR, Jit M. Projecting social contact matrices in 152 countries using contact surveys and demographic data. PLoS Comput Biol. 2017;13:e1005697. doi: 10.1371/journal.pcbi.1005697.

27. Littlewood KJ, Ouwens MJ, Sauboin C, Tehard B, Alain S, Denis F. Cost-effectiveness of routine varicella vaccination using the measles, mumps, rubella and varicella vaccine in France: an economic analysis based on a dynamic transmission model for varicella and herpes zoster. Clin Ther. 2015;37:830-841 e837. doi: 10.1016/j.clinthera.2015.01.006.

28. Briggs AH, Sculpher M, Claxton K. Decision modelling for health economic evaluation. Oxford: Oxford University Press; 2006. 237 p.
