# Supplementary material for: Cost-effectiveness analysis of universal varicella vaccination in Turkey using a dynamic transmission model
Source: PLoS One. 2019 Aug 13;14(8):e0220921. doi: 10.1371/journal.pone.0220921 (PMC6692038; doi:10.1371/journal.pone.0220921)
Supplement: S2 Appendix — (PDF) [file pone.0220921.s002.pdf]

S2 Appendix
Turkey-specific data used in the model

Table A. Original seroprevalence data[a] and the smoothed, monotonically increasing seroprevalence used for Turkey.

Ages (years)	Original Data	Smoothed Data
0 - 1	0.12	0.115
1 - 2	0.28	0.240
2 - 3	0.32	0.359
3 - 4	0.38	0.467
4 - 5	0.565	0.563
5 - 6	0.735	0.644
6 - 7	0.685	0.712
7 - 8	0.85	0.767
8 - 9	0.85	0.811
9 - 10	0.835	0.846
10 - 11	0.85	0.873
11 - 12	0.835	0.894
12 - 13	0.815	0.910
13 - 14	0.94	0.922
14 - 15	0.925	0.931
15 - 20	0.933	0.938
20 - 30	0.944	0.953
30 - 40	0.939	0.960
≥40	0.944	0.960

[a]Original seroprevalence data for ages 0–15 years taken from Gurgoze et al [1] and for ages 15 years and older from Kose et al. [2]

Table B. Age-specific susceptibilities resulting from model calibration to seroprevalence.

Ages (years)	Contact rate
<1	1.03741
1 - 2	1.08054
2 - 3	1.9632
3 - 4	0.756157
4 - 5	1.03684
5 - 6	1.03132
6 - 7	1.05232
7 - 8	1.05666
8 - 9	1.04047
9 - 10	1.00432
10 - 11	1.25408
11 - 12	1.15931
12 - 13	1.04747
13 - 14	0.924795
14 - 15	0.798044
15 - 20	1.93366
20 - 25	1.03897
25 - 30	2.46236
30 - 40	0.302916
≥40	1.03741

Table C. Case fatality rates per 100,000 cases in Turkey.[a]

Age groups (years)	<1	1–5	5–10	10–15	15–45	45–65	≥65
**Natural varicella**	1.04	0.26	0.65	3.12	31.22	322.56	1461.06
**Breakthrough varicella**[b]	0.21	0.05	0.13	0.62	6.24	64.51	292.21

[a]Age group boundaries are closed on the left (boundary is included in the group) and open on the right (boundary is not included in the group)

[b]Assumption that the case fatality rate for breakthrough varicella is one-fifth of the natural varicella case fatality rate based on work by Brisson and Edmunds [3]

Table D. HZ reactivation rates resulting from model calibration.

Ages (years)	<15	15–25	25–35	35–45	45–55	55–65	65–70	70–80	80–90	≥90
Reactivation rate	0.141	0.059	0.035	0.027	0.035	0.061	0.111	0147	396	0.0292

Table E. Age group structure of HZ incidence rate (per 1000 person-years) from published data (original [4]) and averaged HZ data used in the model for ages ≥65 years (adjusted).[a]

**Original age groups**	0–15	15–25	25–35	35–45	45–55	55–65	65–75	75–85	≥85	
HZ incidence	2.39	3.98	3.03	3.88	5.79	9.14	11.77	13.25	10.51	
**Adjusted age groups**	0–15	15–25	25–35	35–45	45–55	55–65	65–70	70–80	80–90	≥90
HZ incidence	2.39	3.98	3.03	3.88	5.79	9.14	11.77	12.51	11.88	10.51

[a]Age group boundaries are closed on the left (boundary is included in the group) and open on the right (boundary is not included in the group)

Table F. Mean direct and indirect treatment costs (2015 Turkish lira, TRY) and health resource utilization by age group for Turkey.

	All	Age group (years)[a]						
**Varicella-related outpatient**		**<1**	**1 - 5**	**5 - 10**	**10 - 15**	**15 - 45**	**45 - 65**	**≥65**
% seeking care[b]	90							
No. of office visits[b]		2	1.5	1	1	1	1	2
Cost per visit[d]	55							
% receiving medications[b]	100							
No. of medications per case	3							
Cost per medication[c]	10.32							
**Varicella-related inpatient**								
% cases hospitalized[b]		25	8	4.2	3.7	4.2	6.5	14.7
No. of hospitalization days		6[c]	7[c]	8[c]	8[c]	7[c]	5	7
Cost per day		138[c]	161[c]	234[c]	217[c]	228	112	112
% receiving medications[e]	0							
No. of medications per case[e]	0							
Cost per medication	0							
**Uncomplicated HZ[g]**								
% seeking care	100							
Cost per case	873							
% receiving medications	0							
No. of medications per case	0							
Cost per medication	0							
**HZ postherpetic neuralgia[g]**								
% seeking care	100							
Cost per case	1883							
% receiving medications	0							
No. of medications per case	0							
Cost per medication	0							
**Varicella workdays lost**								
Outpatient[f]		2.5	2.5	2.5	2.5	2.5	2.5	0
Inpatient[f]		6.8	8.07	8.68	9.36	7.6	6.1	0
1st dose visit[b]	0.5							
2nd dose visit	0.5							
Catch-up dose visit	0.5							
**HZ workdays lost**								
Uncomplicated		2.0	2.0	2.0	2.0	22.7	22.7	22.7

Postherpetic neuralgia		4.6	4.6	4.6	4.6	44.9	44.9	44.9
**Cost**								
Cost per workday missed	55.74							

Unless otherwise indicated, all values are means.

HZ, herpes zoster; No., number.

[a]Age group boundaries are closed on the left (boundary is included in the group) and open on the right (boundary is not included in the group)

[b]According to expert panel of Turkish physicians.

[c]VARICOMP study [5,6]

[d]Social Security system price in Turkey

[e]Assumed to be included in overall hospitalization costs.

[f] Wolfson et al. 2019 [7]

[g]Utilization data, and costs (subsequently converted from MXN to TRY) from Rampakakis et al. 2017 [8]

Table G. All the strategies considered.

Label	Schedule	Exogenous Boosting	HZ disease Impact
1D-EB-HZ	1 dose only	Included	Included
2DS-EB-HZ	2 doses – short interval	Included	Included
2DL-EB-HZ	2 doses – long interval	Included	Included
1D-EB	1 dose only	Included	Excluded
2DS-EB	2 doses – short interval	Included	Excluded
2DL-EB	2 doses – long interval	Included	Excluded
1D	1 dose only	Excluded	Excluded
2DS	2 doses – short interval	Excluded	Excluded
2DL	2 doses – long interval	Excluded	Excluded

EB, exogenous boosting; HZ, herpes zoster

1D, 1-dose; 2DS, 2-dose-short, and 2DL, 2-dose-long vaccination strategies.

Table H. One-way sensitivity input parameters for Turkish input data.

Parameter		Age Groups							
		All	<1	1-5	5-10	10-15	15-45	45-65	65+
% Receiving Outpatient Varicella Care	Base		90	90	90	90	90	90	90
	Lower Bound		72	72	72	72	72	72	72
	Upper Bound		100	100	100	100	100	100	100
% Varicella Cases Hospitalized	Base		25	8	4.2	3.7	4.2	6.5	14.7
	Lower Bound		20	6.4	3.36	2.96	3.36	5.2	11.76
	Upper Bound		30	9.6	5.04	4.44	5.04	7.8	17.64
% Varicella Outpatient Receiving Medication	Base		100	100	100	100	100	100	100
	Lower Bound		80	80	80	80	80	80	80
	Upper Bound		100	100	100	100	100	100	100
% HZ non-PHN Receiving Care	Base		100	100	100	100	100	100	100
	Lower Bound		80	80	80	80	80	80	80
	Upper Bound		100	100	100	100	100	100	100
% PHN Receiving Care	Base		100	100	100	100	100	100	100
	Lower Bound		80	80	80	80	80	80	80
	Upper Bound		100	100	100	100	100	100	100
Cost of Outpatient Visit Varicella	Base		55	55	55	55	55	55	55
	Lower Bound		44	44	44	44	44	44	44
	Upper Bound		66	66	66	66	66	66	66
Cost of Varicella Hospital Day	Base		138.4	161.2	234	216.7	228.4	111.5	111.5
	Lower Bound		110.7	129	187.2	173.4	182.7	89.2	89.2
	Upper Bound		166.1	193.4	280.8	260	274.1	133.8	133.8
Average Cost Outpatient Varicella Drugs	Base	10.32							
	Lower Bound	8.26							
	Upper Bound	12.39							
Cost HZ care	Base		873.3	873.3	873.3	873.3	873.3	873.3	873.3
	Lower Bound		698.6	698.6	698.6	698.6	698.6	698.6	698.6
	Upper Bound		1048	1048	1048	1048	1048	1048	1048
Cost PHN Care	Base		1883	1883	1883	1883	1883	1883	1883
	Lower Bound		1507	1507	1507	1507	1507	1507	1507

Parameter		Age Groups							
	Upper Bound		2260	2260	2260	2260	2260	2260	2260
# Outpatient Visits Varicella	Base		2	1.5	1	1	1	1	2
	Lower Bound		1.6	1.2	0.8	0.8	0.8	0.8	1.6
	Upper Bound		2.4	1.8	1.2	1.2	1.2	1.2	2.4
# Hospital Days Varicella	Base		5.8	7.07	7.68	8.36	6.64	5.13	7
	Lower Bound		4.64	5.656	6.144	6.688	5.312	4.104	5.6
	Upper Bound		6.96	8.484	9.216	10.03	7.968	6.156	8.4
# of Outpatient Medications Varicella	Base		3	3	3	3	3	3	3
	Lower Bound		2.4	2.4	2.4	2.4	2.4	2.4	2.4
	Upper Bound		3.6	3.6	3.6	3.6	3.6	3.6	3.6
Utilities Breakthrough Varicella			<15	15+					
	Base		0.905	0.865					
	Lower Bound		0.724	0.692					
	Upper Bound		1	1					
Utilities Natural Varicella	Base		0.81	0.73					
	Lower Bound		0.648	0.584					
	Upper Bound		0.972	0.876					
Utilities Breakthrough HZ	Base	0.73							
	Lower Bound	0.584							
	Upper Bound	0.876							
Utilities PHN	Base	0.671							
	Lower Bound	0.5368							
	Upper Bound	0.8052							
Utilities HZ	Base	0.73							
	Lower Bound	0.584							
	Upper Bound	0.876							
1st Dose Duration of Protection	Base	25							
	Lower Bound	20							
	Upper Bound	30							
2nd Dose Duration of Protection	Base	77							
	Lower Bound	61.6							

Parameter		Age Groups
	Upper Bound	92.4
Duration of HZ vaccine immunity	Base	4.3
	Lower Bound	3.44
	Upper Bound	5.16
1st Dose % Take	Base	100
	Lower Bound	80
	Upper Bound	100
2nd Dose % Take	Base	100
	Lower Bound	80
	Upper Bound	100
Varicella Susceptibility after 1st Dose Waning	Base	100
	Lower Bound	80
	Upper Bound	100
Varicella Susceptibility after 2nd Dose Waning	Base	100
	Lower Bound	80
	Upper Bound	100
Rate of Waning to HZ Susceptible from 1st Dose Protected	Base	0.04
	Lower Bound	0.032
	Upper Bound	0.048
Rate of Waning to HZ Susceptible from 2nd Dose Protected	Base	0.0130
	Lower Bound	0.0104
	Upper Bound	0.0156
% Successful Vaccinated	Base	96
	Lower Bound	76.8
	Upper Bound	100
Probability of boosting to HZ protected	Base	100
	Lower Bound	80

Parameter		Age Groups							
from 1st Dose Protected	Upper Bound	100							
Probability of boosting to HZ protected from 2nd Dose Protected	Base	100							
	Lower Bound	80							
	Upper Bound	100							
Varicella Vaccine Dose Cost	Base	60.55							
	Lower Bound	48.44							
	Upper Bound	72.66							
Cost of Missed Workday	Base	55.74							
	Lower Bound	44.59							
	Upper Bound	66.89							
# Workdays Lost Outpatient Varicella Care			<1	1-5	5-10	10-15	15-45	45-65	65+
	Base		2.5	2.5	2.5	2.5	2.5	2.5	0
	Lower Bound		2	2	2	2	2	2	0
	Upper Bound		3	3	3	3	3	3	0
# Workdays Lost Inpatient Varicella Care	Base		6.8	8.07	8.68	9.36	7.6	6.1	0
	Lower Bound		5.44	6.456	6.944	7.488	6.08	4.88	0
	Upper Bound		8.16	9.684	10.42	11.23	9.12	7.32	0
# Workdays Lost HZ	Base		1.982	1.982	1.982	1.982	22.71	22.71	22.71
	Lower Bound		1.586	1.586	1.586	1.586	18.16	18.16	18.16
	Upper Bound		2.379	2.379	2.379	2.379	27.25	27.25	27.25
# Workdays Lost PHN	Base		4.575	4.575	4.575	4.575	44.87	44.87	44.87
	Lower Bound		3.66	3.66	3.66	3.66	35.89	35.89	35.89
	Upper Bound		5.49	5.49	5.49	5.49	53.84	53.84	53.84
# Workdays Lost Vaccination	Base	0.5							
	Lower Bound	0.4							
	Upper Bound	0.6							
1st Dose Coverage	Base	95							
	Lower Bound	75							
	Upper Bound	95							
2nd Dose									

Parameter		Age Groups	
Coverage	Base	90	
	Lower Bound	70	
	Upper Bound	95	
Reactivation Rate Vaccine Adjustment	Base	0.1667	
	Lower Bound	0.0833	
	Upper Bound	0.25	

HZ, herpes zoster; PHN, postherpetic neuralgia.

References

1. Gurgoze MK, Yilmaz E, Godekmerdan A, Akca Z, Dogan Y, Akarsu S, et al. Seroprevalence of mumps, varicella and rubella antibodies in children 1-16 years of age in eastern Turkey. Turk J Pediatr. 2006;48:185-188.
2. Kose S, Mandiracioglu A, Senger SS, Ulu Y, Cavdar G, Gol B, et al. Seroprevalence of varicella-zoster virus in the prevaccine era: a population-based study in Izmir, Turkey. J Infect Public Health. 2013;6:115-119. doi: 10.1016/j.jiph.2012.10.003.
3. Brisson M, Edmunds WJ. Varicella vaccination in England and Wales: cost-utility analysis. Arch Dis Child. 2003;88:862-869.
4. Weitzman D, Shavit O, Stein M, Cohen R, Chodick G, Shalev V. A population based study of the epidemiology of Herpes Zoster and its complications. J Infect. 2013;67:463-469. doi: 10.1016/j.jinf.2013.06.016.
5. Dinleyici EC, Kurugol Z, Turel O, Hatipoglu N, Devrim I, Agin H, et al. The epidemiology and economic impact of varicella-related hospitalizations in Turkey from 2008 to 2010: a nationwide survey during the pre-vaccine era (VARICOMP study). Eur J Pediatr. 2012;171:817-825. doi: 10.1007/s00431-011-1650-z.
6. Dinleyici EC, Kurugol Z, Kara A, Tezer H, Tas MA, Guler E, et al. Children with breakthrough varicella infection requiring hospitalization in Turkey (VARICOMP Study 2008-2013). Vaccine. 2015;33:3983-3987. doi: 10.1016/j.vaccine.2015.06.029.
7. Wolfson LJ, Castillo ME, Giglio N, Meszner Z, Molnar Z, Vazquez M, et al. Varicella healthcare resource utilization in middle income countries: a pooled analysis of the multi-country MARVEL study in Latin America & Europe. Hum Vaccin Immunother. 2019;15:932-941. doi: 10.1080/21645515.2018.1559687.
8. Rampakakis E, Pollock C, Vujacich C, Toniolo Neto J, Ortiz Covarrubias A, Monsanto H, et al. Economic burden of herpes zoster ("culebrilla") in Latin America. Int J Infect Dis. 2017;58:22-26. doi: 10.1016/j.ijid.2017.02.021.
