# Supplementary figures and images for: Cost-effectiveness analysis of universal varicella vaccination in Turkey using a dynamic transmission model
Source: PLoS One. 2019 Aug 13;14(8):e0220921. doi: 10.1371/journal.pone.0220921 (PMC6692038; doi:10.1371/journal.pone.0220921)

**S1 Fig. Model calibration: modeled HZ incidence rate and the adjusted observed incidence rate.**

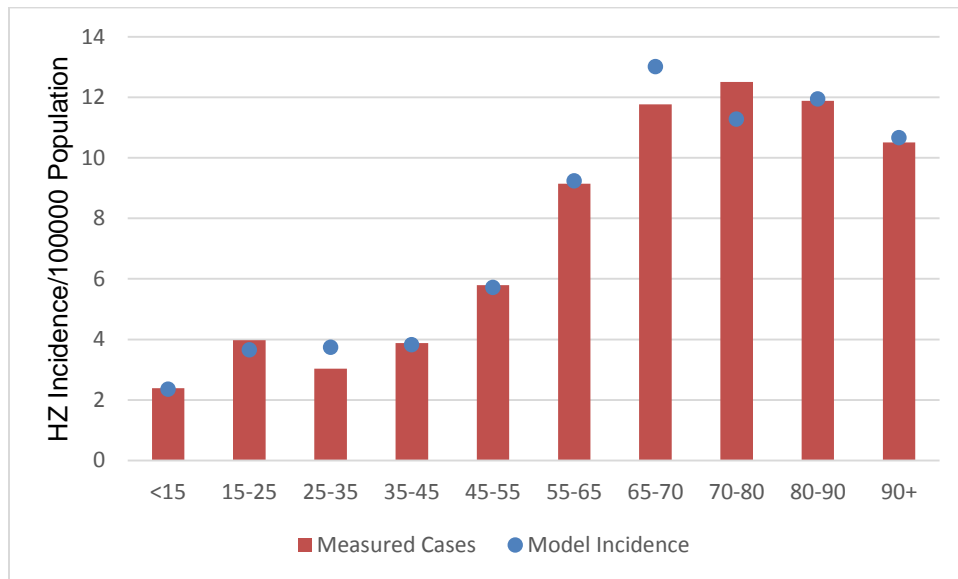

Supplement: S1 Fig — (PDF) [file pone.0220921.s003.pdf]
