# Supplementary material for: Cost-effectiveness analysis of universal varicella vaccination in Turkey using a dynamic transmission model
Source: PLoS One. 2019 Aug 13;14(8):e0220921. doi: 10.1371/journal.pone.0220921 (PMC6692038; doi:10.1371/journal.pone.0220921)

S2 Fig. Age distribution of varicella cases by vaccination strategy: percentage of total varicella incidence at 100 years.

1D, 1-dose; 2DS, 2-dose-short, and 2DL, 2-dose-long vaccination strategies.

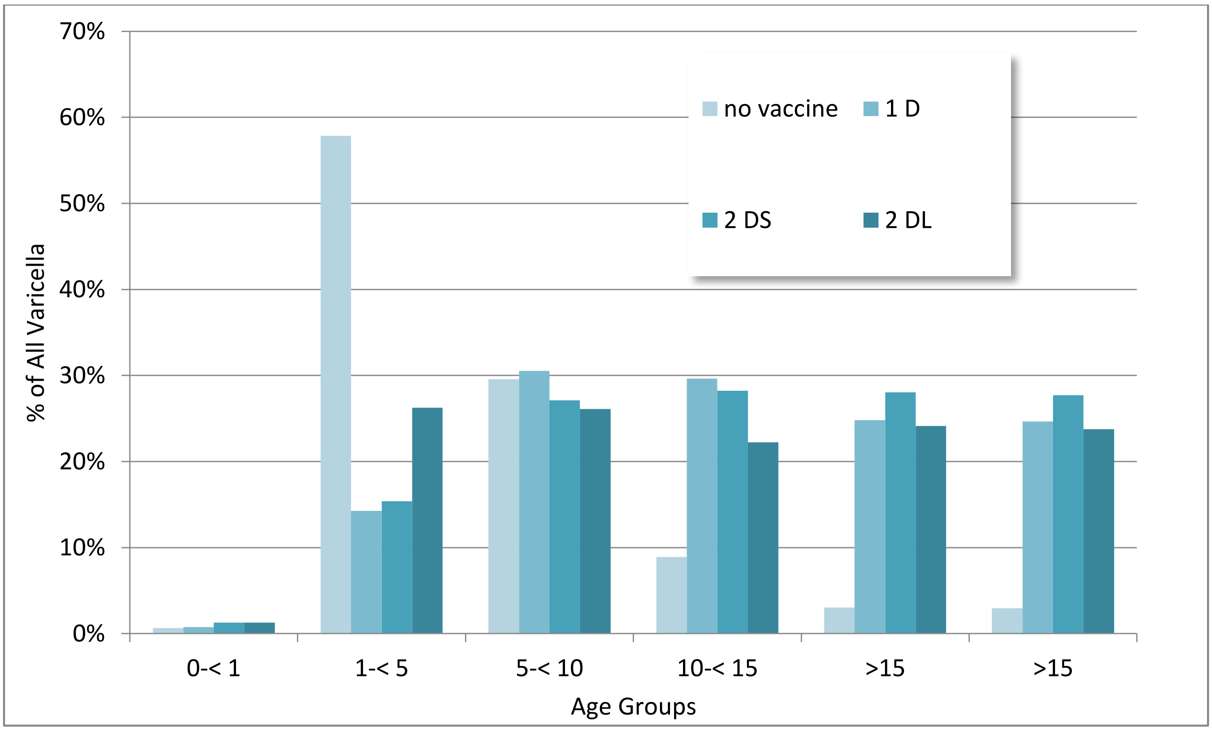

Supplement: S2 Fig — (PDF) [file pone.0220921.s004.pdf]
