# Supplementary material for: Cost-effectiveness analysis of universal varicella vaccination in Turkey using a dynamic transmission model
Source: PLoS One. 2019 Aug 13;14(8):e0220921. doi: 10.1371/journal.pone.0220921 (PMC6692038; doi:10.1371/journal.pone.0220921)

S3 Fig. Age-specific reductions in varicella incidence at 5, 10, and 25 years after start of vaccination using three vaccination strategies and three types of vaccines of differing effectiveness.

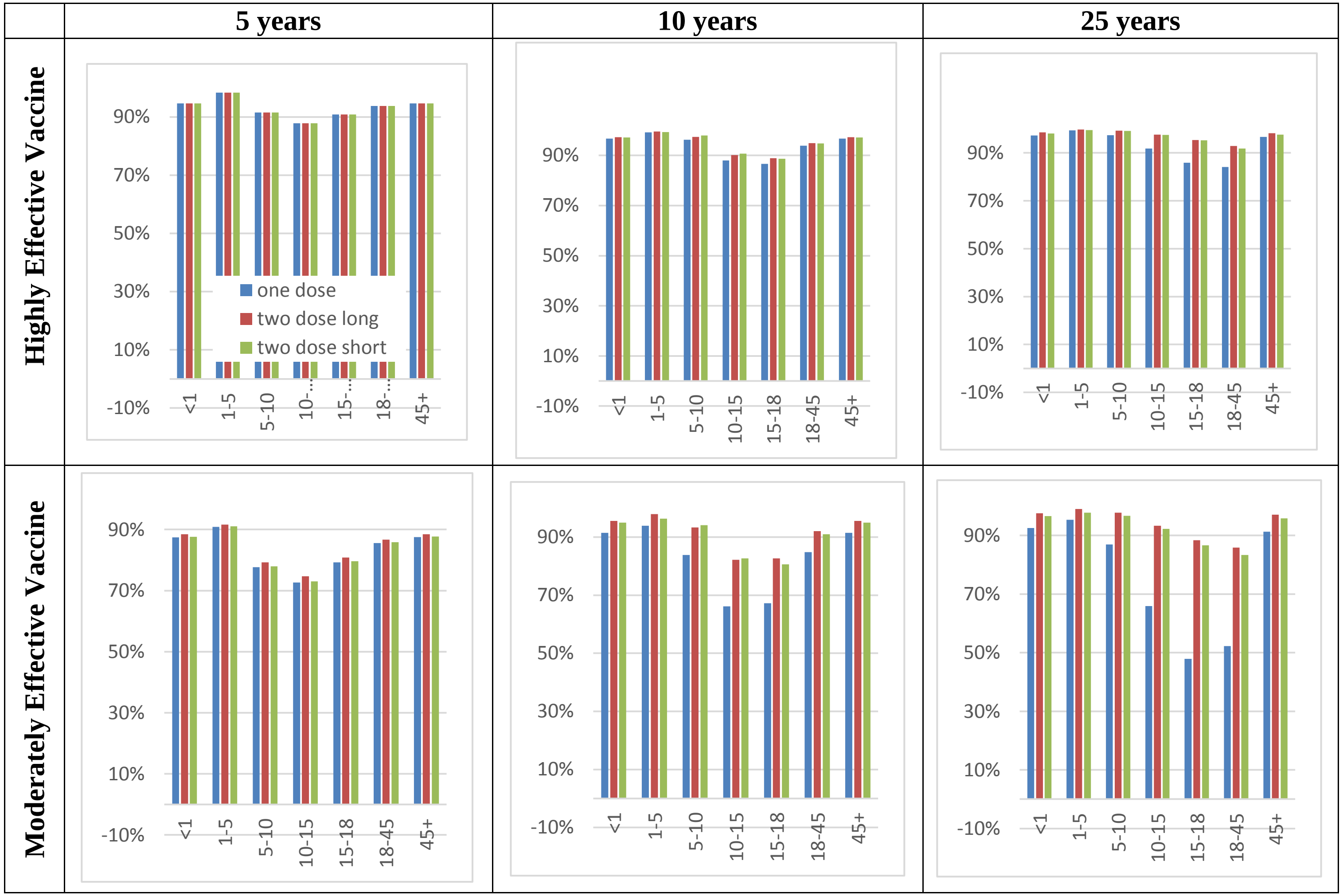

Weakly Effective Vaccine

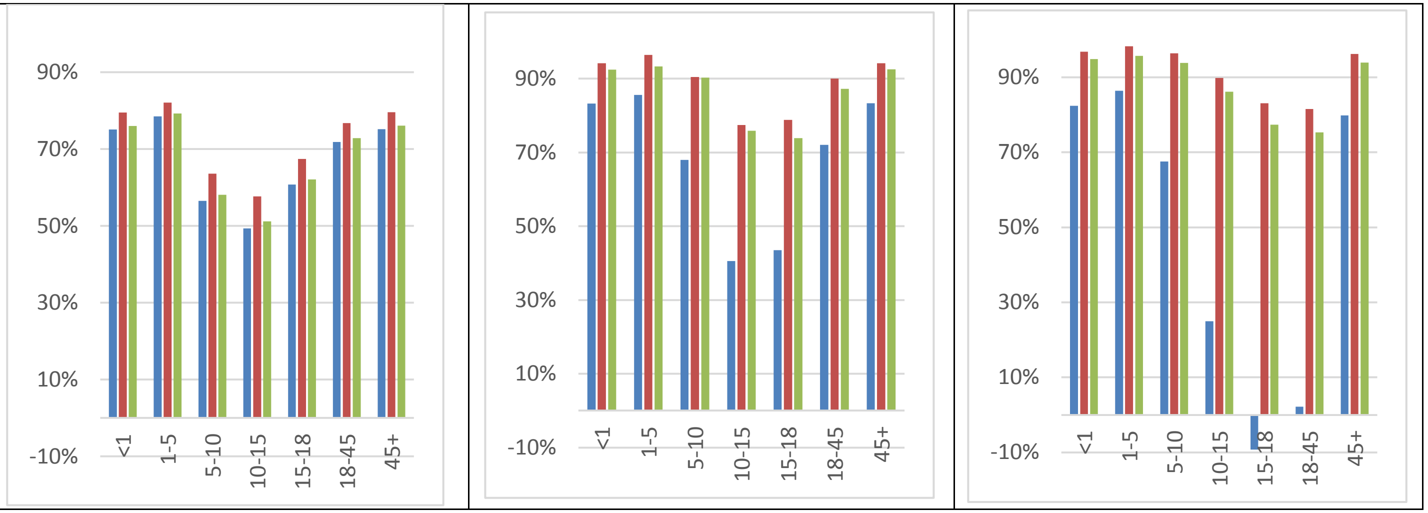

Supplement: S3 Fig — (PDF) [file pone.0220921.s005.pdf]
