# Supplementary material for: Cost-effectiveness analysis of universal varicella vaccination in Turkey using a dynamic transmission model
Source: PLoS One. 2019 Aug 13;14(8):e0220921. doi: 10.1371/journal.pone.0220921 (PMC6692038; doi:10.1371/journal.pone.0220921)

S4 Fig. Distribution of costs over time by varicella vaccine type and vaccination strategy.

1D, 1-dose; 2D-S, 2-dose-short, and 2D-L, 2-dose-long vaccination strategies; HEV, highly effective vaccine; HZ, herpes zoster; VZV, varicella; WEV, weakly effective vaccine.

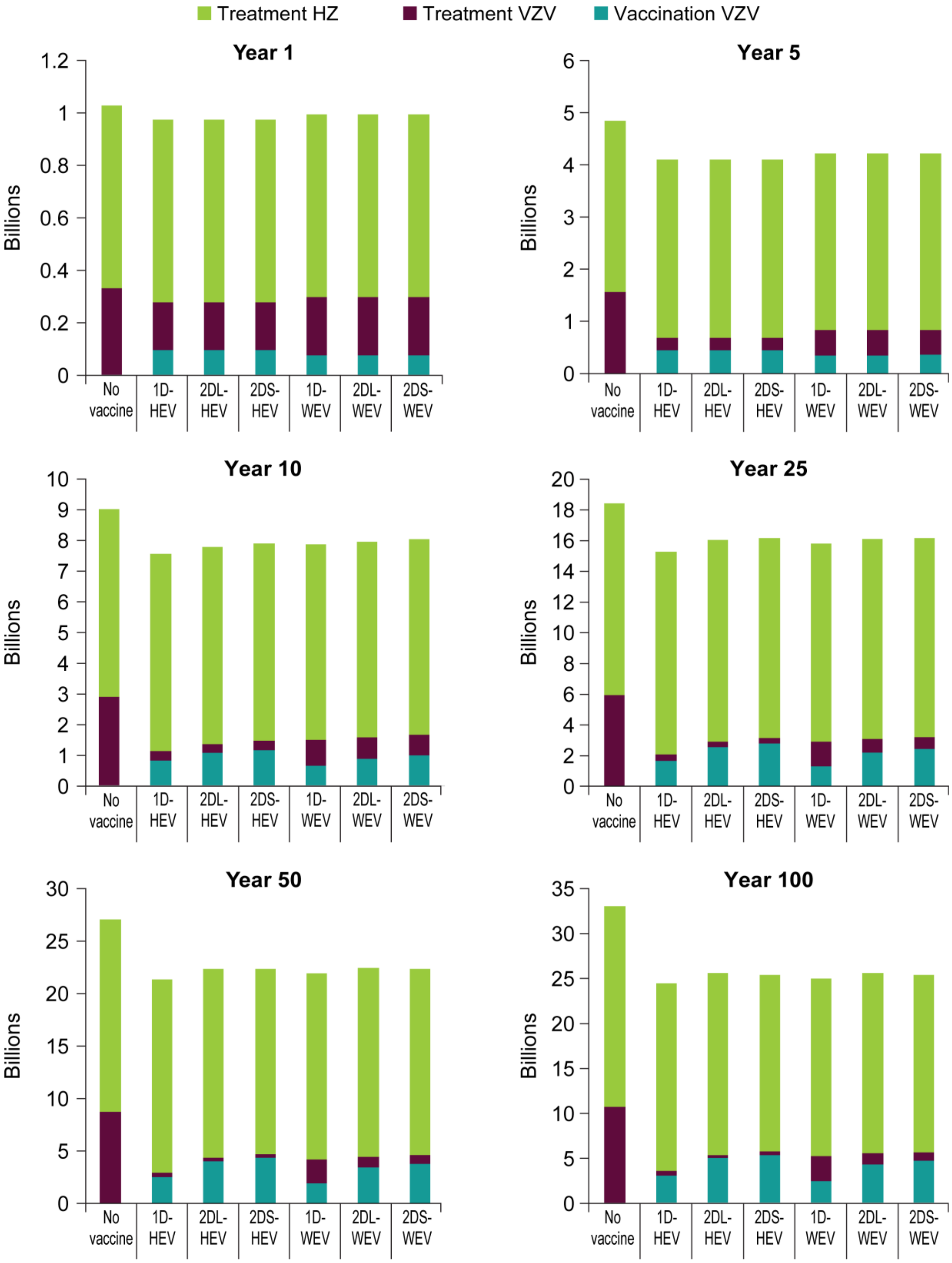

Supplement: S4 Fig — (PDF) [file pone.0220921.s006.pdf]
