# Supplementary material for: Cost-effectiveness analysis of universal varicella vaccination in Turkey using a dynamic transmission model
Source: PLoS One. 2019 Aug 13;14(8):e0220921. doi: 10.1371/journal.pone.0220921 (PMC6692038; doi:10.1371/journal.pone.0220921)

S5 Fig. Age distribution of HZ cases by vaccination strategy. (A) with exogenous boosting and (B) without exogenous boosting: percentage of total HZ incidence at 100 years. 1D, 1-dose; 2DS, 2-dose-short, and 2DL, 2-dose-long vaccination strategies.

(A)

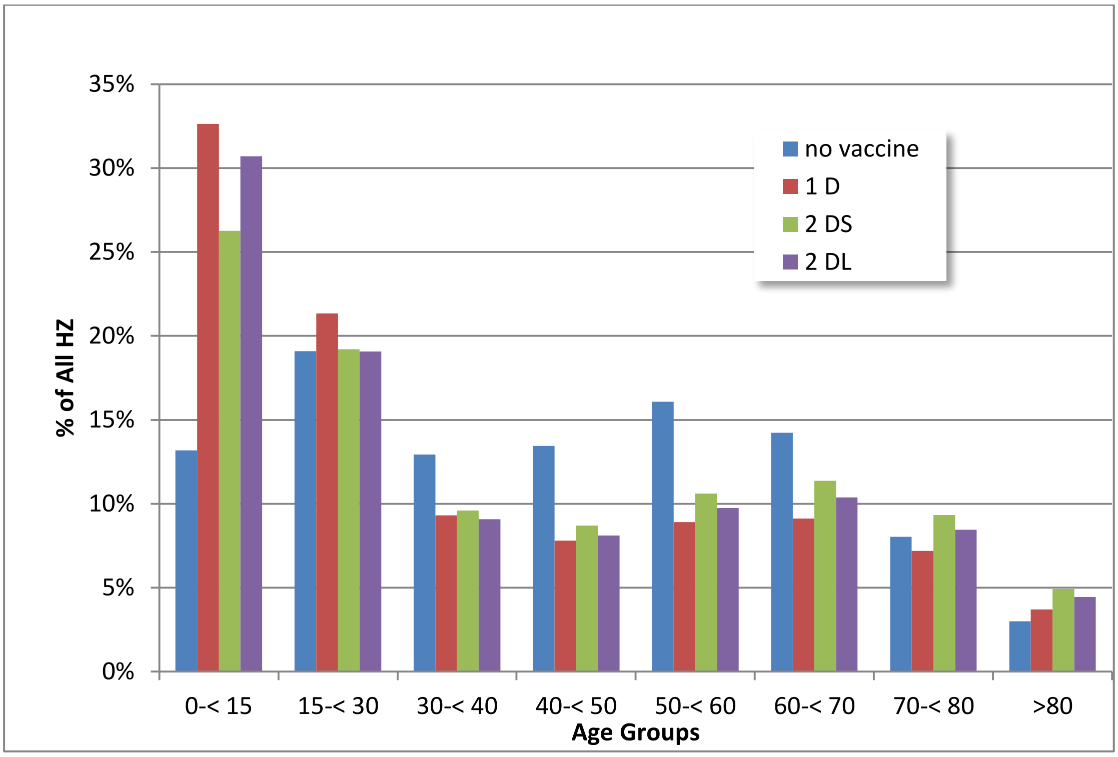

(B)

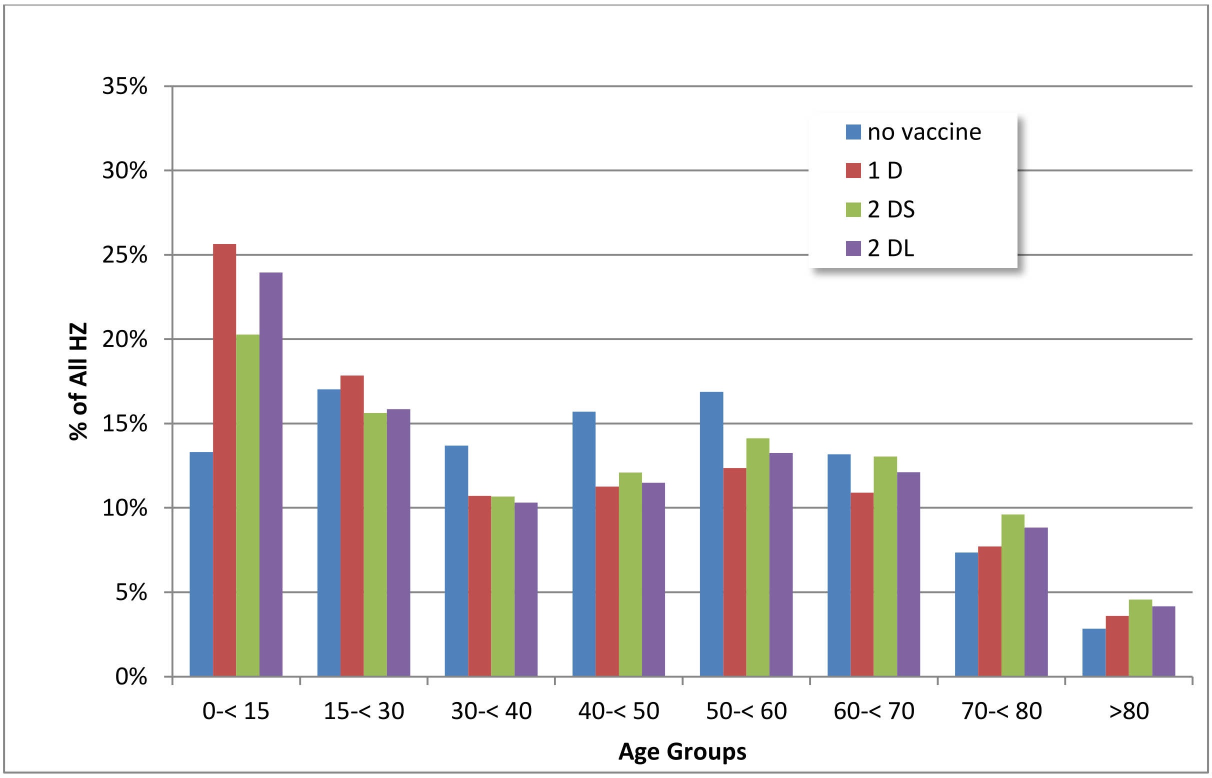

Supplement: S5 Fig — (A) with exogenous boosting and (B) without exogenous boosting: percentage of total HZ incidence at 100 years. (PDF) [file pone.0220921.s007.pdf]
