# Supplementary material for: Cost-effectiveness analysis of universal varicella vaccination in Turkey using a dynamic transmission model
Source: PLoS One. 2019 Aug 13;14(8):e0220921. doi: 10.1371/journal.pone.0220921 (PMC6692038; doi:10.1371/journal.pone.0220921)
Supplement: S1 Table — (PDF) [file pone.0220921.s008.pdf]

S1 Table. Cumulative results for varicella disease in Turkey using 1-dose (1D), 2-dose-short (2DS), or 2-dose-long (2DL) varicella vaccination strategy over 1–100 years.

Cumulative results			
**% Cumulative varicella-related deaths avoided**			
**Timeline**	**1D**	**2DS**	**2DL**
1 year	40%	40%	40%
5 years	82%	82%	82%
10 years	88%	88%	88%
25 years	92%	93%	93%
50 years	93%	96%	95%
100 years	94%	97%	96%
**% Cumulative varicella cases avoided**			
1 year	46%	45%	46%
5 years	85%	85%	85%
10 years	90%	91%	91%
25 years	95%	96%	96%
50 years	96%	97%	97%
100 years	97%	98%	98%
**% Cumulative congenital varicella cases avoided**			
1 year	33%	33%	33%
5 years	81%	81%	81%
10 years	87%	87%	87%
25 years	89%	91%	91%
50 years	87%	93%	92%
100 years	86%	94%	93%
