# Supplementary material for: Cost-effectiveness analysis of universal varicella vaccination in Turkey using a dynamic transmission model
Source: PLoS One. 2019 Aug 13;14(8):e0220921. doi: 10.1371/journal.pone.0220921 (PMC6692038; doi:10.1371/journal.pone.0220921)
Supplement: S2 Table — (PDF) [file pone.0220921.s009.pdf]

S2 Table. Direct, indirect, and total costs for Turkey using one-dose (1D), 2-dose-short (2DS), or 2-dose-long (2DL) varicella vaccination strategies with a highly effective vaccine, with and without including exogenous boosting and with and without including HZ-related costs. Costs are per capita and calculated as cumulative costs after 100 years in billions TRY, discounted at 3% annual rate.

Cost Type	No Vaccine	Vaccination strategy		
		1-Dose	2-Dose-Short	2-Dose-Long
With EB, including HZ-related costs				
Treatment costs				
Direct costs	15.8247	10.9703	10.1687	10.6012
Indirect costs	17.2764	10.4018	9.8885	10.0907
Total costs	33.1011	21.3721	20.0573	20.6919
Vaccine costs	-	-	-	-
Direct costs	-	2.4458	4.3077	3.9790
Indirect costs	-	0.6081	1.0710	0.9893
Total costs	-	3.0538	5.3786	4.9682
Total costs (direct + indirect)	33.1011	24.4259	25.4359	25.6601
With No EB, including HZ Costs				
Treatment costs				
Direct costs	16.0090	9.4855	9.0079	9.2636
Indirect costs	17.7721	9.5769	9.2381	9.3786
Total costs	33.7811	19.0624	18.2461	18.6422
Vaccine costs	-	-	-	-
Direct costs	-	2.4459	4.3120	3.9936
Indirect costs	-	0.6081	1.0721	0.9929
Total costs	-	3.0541	5.3841	4.9865
Total costs (direct + indirect)	33.7811	22.1164	23.6301	23.6287
With EB, excluding HZ-related costs				
Treatment costs				
Direct costs	4.6607	0.2015	0.1523	0.1592
Indirect costs	6.0005	0.2725	0.2037	0.2120
Total costs	10.6613	0.4740	0.3561	0.3712
Vaccine costs	-	-	-	-
Direct costs	-	2.4458	4.3077	3.9790
Indirect costs	-	0.6081	1.0710	0.9893
Total costs	-	3.0538	5.3786	4.9682
Total costs (direct + indirect)	10.6613	3.5278	5.7347	5.3394
With no EB, excluding HZ-related costs				
Treatment costs				
Direct costs	4.6607	0.1605	0.1319	0.1355
Indirect costs	6.0005	0.2153	0.1753	0.1796
Total costs	10.6613	0.3757	0.3072	0.3151
Vaccine costs	-	-	-	-
Direct costs	-	2.4459	4.3120	3.9936
Indirect costs	-	0.6081	1.0721	0.9929
Total costs	-	3.0541	5.3841	4.9865
Total costs (direct + indirect)	10.6613	3.4298	5.6913	5.3016
